# Remodeling of Stromal Cells and Immune Landscape in Microenvironment During Tumor Progression

**DOI:** 10.3389/fonc.2021.596798

**Published:** 2021-03-08

**Authors:** Leena Arora, Durba Pal

**Affiliations:** Tissue Engineering and Regenerative Medicine Lab, Indian Institute of Technology Ropar, Rupnagar, India

**Keywords:** tumor microenvironment, stromal cell, immune cell, tumor progression, non coding RNAs

## Abstract

The molecular understanding of carcinogenesis and tumor progression rests in intra and inter-tumoral heterogeneity. Solid tumors confined with vast diversity of genetic abnormalities, epigenetic modifications, and environmental cues that differ at each stage from tumor initiation, progression, and metastasis. Complexity within tumors studied by conventional molecular techniques fails to identify different subclasses in stromal and immune cells in individuals and that affects immunotherapies. Here we focus on diversity of stromal cell population and immune inhabitants, whose subtypes create the complexity of tumor microenvironment (TME), leading primary tumors towards advanced-stage cancers. Recent advances in single-cell sequencing (epitope profiling) approach circumscribes phenotypic markers, molecular pathways, and evolutionary trajectories of an individual cell. We discussed the current knowledge of stromal and immune cell subclasses at different stages of cancer development with the regulatory role of non-coding RNAs. Finally, we reported the current therapeutic options in immunotherapies, advances in therapies targeting heterogeneity, and possible outcomes.

## Introduction

“What is it that determines what organs shall suffer in the case of disseminated cancer”? (1). The answer to this question proposed by Stephan Paget way back in 1889 led oncologists to study different perspectives of growing tumors. Oncology is now branched to multiple disciplines starting from early studies on genomic alterations, chromosomal aberrations, altered signaling, and cell plasticity. Cancers are more than “just a disease of the genome” and hint clinicians to study various aspects of tumor biology. During development, cancer cells undergo stochastic genetic and epigenetic alterations leading to molecular and phenotypic differences that have implications in heterogeneity, forcing the construction of their niche (2). Tumors have multiple non-cancerous regenerative cell types that eventually differentiate into various tumor supporting cells (3). Lineage plasticity enables cancer cells to adapt to changing environmental conditions like aberrant vasculature, low oxygen tension, high metabolic rates, and low pH. It is the ability of a non-cancerous/cancerous cell to differentiate into other cell types of distinct lineage (4). Embryonic stem cells (ESCs), mesenchymal stem cells (MSCs), and adult stem cells (ADCs) within the TME define routes to cell plasticity and are the known originators of other cell types like cancer-associated fibroblasts (CAFs), tumor endothelial cells (TECs), tumor-associated macrophages (TAMs) to name a few. These stem cells have the potential to differentiate into a broad spectrum of cell types; however, the activation of pathways that control stem cell is poorly understood (5). Studies on ESCs and pathway analysis would help us to understand the developmental plasticity in TME. Development of fundamental methods to understand the role of ESCs in tumors include a) development of organoid models from patient-derived samples, b) culture of ESCs in the artificial stromal environment, and c) study of activation pathways that derive stem cell characteristics. Tumor organoids lead cancer research to study stromal and immune cell interaction in TME but have certain limitations. Patient-derived tumor organoids do not always recreate tumor tissue architecture as they are anchorage-dependent (requires basement membrane).The addition of extracellular matrix may not be a passive bystander, but the biological consequences of this are unexplored. Phenotypic and genotypic variations from organoid to organoid may affect the experimental reproducibility and enhance false-positive results. Potential contaminants like normal epithelial cells cannot be ignored and hence affect pathway studies in tumors. Lineage-specific transcription factors that are known to govern embryonic cell fate specifications also plays a role in characterizing cancer subtypes (6).

There are several mechanisms of cellular plasticity. Differentiation is the most common route by which a pluripotent stem cell evolves into a mature cell type (7). Contrarily, de-differentiation reverts the terminally differentiated cancer cells to a less differentiated stage within its lineage. Trans-differentiation is another such route that takes de-differentiation a step further to a point where it delineates and differentiates to other cell types (8). Reprogramming is a different mechanism where a differentiated cell reverts to its pluripotent source to adapt to any other cell type (9). Reprogramming is a less explored area in TME but has a huge potential in regenerative medicine. It outlines the structure for establishing different cell types and promotes the development of stromal cells like epithelial cells, CAFs, TECs, pericytes, and immune cells, including monocytes, TAMs, neutrophils, NK cells, B cells, T cells (10). These cell types also differ phenotypically and genetically as the tumor progresses to advanced stage (11).

Non-coding RNAs function as significant players in post-transcriptional gene regulation within diverse cell types. Cancers are primarily regulated by a different set of non-coding RNAs, classified according to their sizes (12). MicroRNAs (miRNAs), piwi-interacting RNAs (piRNAs), and small interfering RNAs (siRNAs) are generated from precursor molecules. Long non-coding RNAs (lncRNAs) are higher than 200 nucleotides in length produced from intergeneric regions. Circular RNAs (circRNAs) are a class of lncRNAs generated by a process called back-splicing, wherein downstream exons are spliced to upstream exons in the reverse order that acts as miRNA sponges as well (13). Increased sequencing depth and RNA profiling strategies have identified varied sets of non-coding RNAs including large intergenic non-coding RNAs (lincRNAs), intron-derived small nucleolar lincRNAs (sno-lincRNAs), enhancer RNAs (eRNAs), along with already known miRNAs, piRNAs, however, functional roles of many are still unknown in cancers (14). miRNAs, lncRNAs, and circRNAs regulate cancer stem cell characteristics, epithelial-mesenchymal transitions (EMTs), and vice-versa by epigenetic and transcriptional modification (15). The role of non-coding RNA players in regulating stromal and immune cell diversity within TME is specified in the respective sections.

## Stromal Cell Heterogeneity

TME is remarkably a complex ecosystem with a heterogeneous population of cancer cells and the associated stroma, which drives tumor initiation and growth. Stromal cells, extracellular matrix, paracrine signaling molecules (chemokines, cytokines, and growth factors), and immune landscape formulates TME (16). Interactions within genetically altered cancer cells and stromal cells regulate hallmarks of cancer, such as replicative potentiality, sustained angiogenesis, invasion, and metastasis (17). Understanding cell heterogeneity in TME determines which stromal cells have the potential to contribute to tumor development and progression (18). Intra-tumoral heterogeneity within varied tumor types clinically fails/minimally adopts the given therapeutics and is one of the critical reasons for post-treatment immune-modulatory microenvironment and tumor relapse. Therefore, understanding each component and related phenotypic distinctions of this stromal population would help to predict the difference in primary and advanced tumor cell subtypes, genomic co-relations within subtypes, population-specific markers, molecular/cellular pathways governing developmental origins and differentiation, the source of antitumor/inflammatory cells, and modifiers of immune microenvironment. Stromal cells are comparatively fewer than epithelial and immune cells (19). Single-cell RNA sequencing (scRNAseq) gives a comprehensive blueprint of stromal and immune cell subtypes and analyses differentiation dynamics. It identifies rare cell populations and transcriptome responses in specific tumor conditions in individual or collective tumor samples. Advances in this technology enable studies on genetic and non-genetic tumor mechanisms, microenvironmental cues, cell-cell interactions, developmental pathways, rare tumor subpopulations, and investigation of non-responders to cancer treatments (20). Not only for tumor assessments, but single-cell sequencing is also beneficial for developing next-generation cell-based therapies. Its efficiency in providing high-resolution genome-wide molecular readouts allows the characterization of tumor samples at a larger scale, identifying new targets for drug development based on repressed or activated gene expressions (21).

## Diversity in Tumor Remodeling CAFs and Distinct Gene Programs

**Figure 1:**
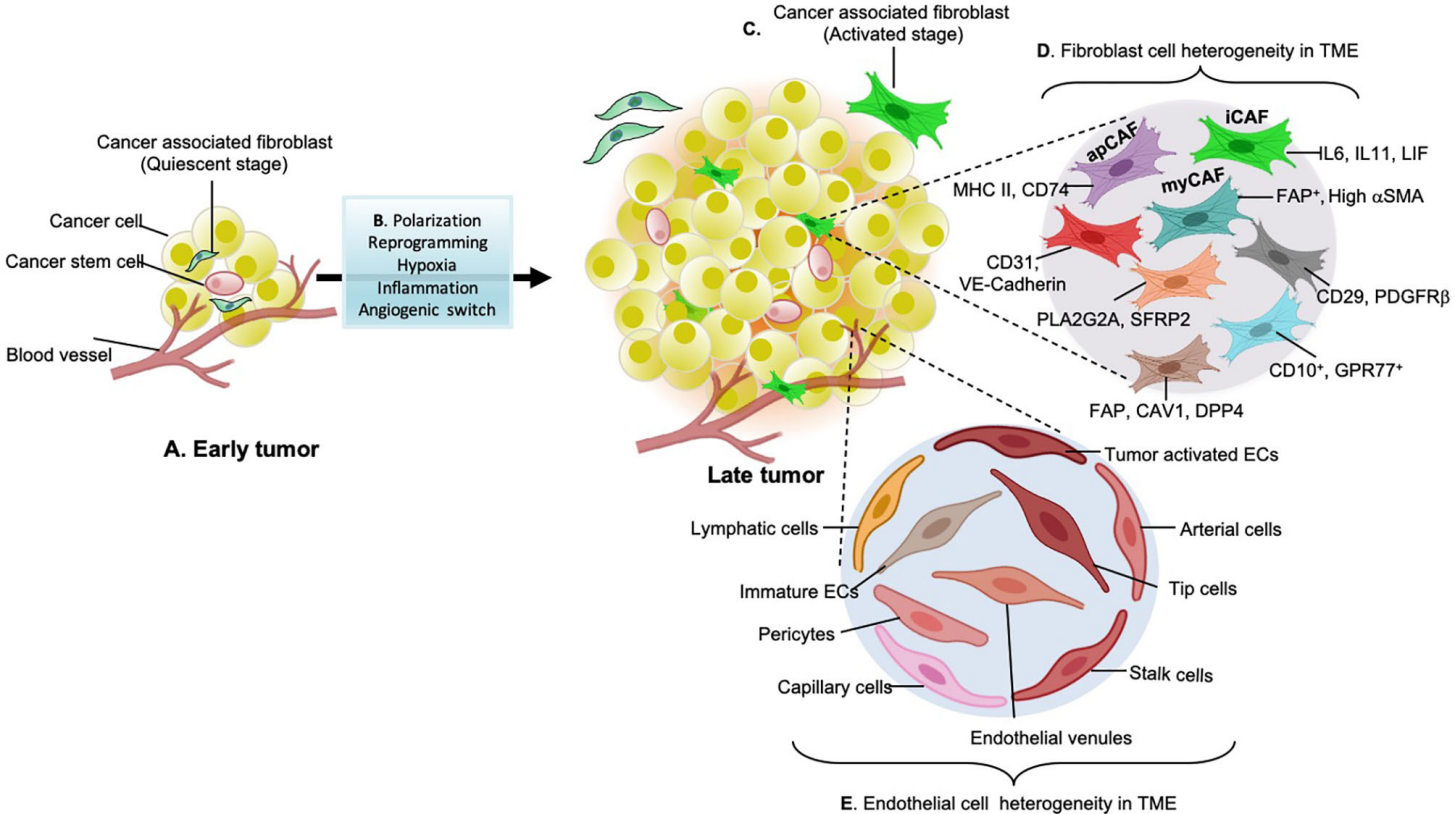
Stromal cell types in early and late-stage tumors. **(A)** early tumors consist of non-aggressive quiescent fibroblasts in the Tumor microenvironment (TME). **(B)** Factors such as hypoxia lead to tumor inflammation and trigger environmental clues that participate in the paracrine signaling loop (cytokines, chemokines, growth factors) and angiogenic switches (VEGF, PDGF-b, FGF, and EGF) which causes stromal cell polarization or reprogramming. Together all these factors are responsible for the diversification of stromal cell types during cancer development. **(C)** Cytokines growth factors such as TGF-b, IL-6 released from tumor cells activate quiescent fibroblasts. **(D)** Activated fibroblasts further reprogram to secretory phenotypes specialized in ECM remodeling and immuno-modulation. These fibroblasts have enhanced proliferative and matrix secreting (desmoplasia) capabilities. Some of these aggressive cancer-associated fibroblasts (CAF) subtypes found in different types of cancers include antigen-presenting CAFs (apCAFs), inflammatory CAFs (iCAFs) and myofibroblastic CAFs (myCAFs). These subtypes are explored through single-cell RNA sequencing transcriptomics and are named according to the secretory factors and roles which these phenotypes play in the TME. **(E)** Different types of endothelial cells found in tumor tissues: common endothelial cell types found in core or adjacent tumor areas are known as tumor endothelial cells. Apart from conventional tip and stalk cells, various subpopulations have been identified that express gene signatures related to the basement membrane breaching (triggering metastasis), immune cell recruitment, and immuno-modulation (causing immunosuppressive TME). Identifying such angiogenic candidates with activated angiogenic transcription factors and enzymes responsible for angiogenesis will be a potential source to develop anti-angiogenic strategies.

CAFs are the major stromal component of many solid tumors and are well known phenotypic remodelers of the stromal environment (22). They constitute a diverse cell population, but the extent of heterogeneity is scarcely explored. Based on different expression signatures, fibroblasts are subdivided into 1) Quiescent fibroblasts: less tumorigenic and primarily found in non-malignant samples and 2) myofibroblasts/CAFs that promote tumors causing tumor resistance, relapse, and strongly enriched in malignant or metastatic tumors (23). They secrete a unique repertoire of collagens and elastins, maintaining the extracellular matrix, thus characterizes desmoplasia (23). Quiescent fibroblasts secrete low levels of collagens, specifically COL13A1 and COL14A1 and high levels of elastins. Myofibroblasts exclusively originate from tumor tissues and are mostly enriched in collagens and low elastins (24). Myofibroblast subtypes have different activation mechanisms such as TGF-β1, IL-11 stimulation, and IL-1β, IL-6 treatment that induces upregulation of inflammatory fibroblast marker genes (iCAFs) (25). CAF specific markers of identification include α smooth muscle actin (αSMA) (also known as ACTA2), desmin, S100A4, Fibroblast activated protein (FAP), express pro-inflammatory cytokine arrays like IL-1β, IL-6, IL-8, TGF-β, CXCL12, and collagen (18). Fibroblastic reticular cells are another immunologically specialized myofibroblasts that are known to attract immune cells within lymph nodes. These fibroblasts generate ECM for the transit of potential antigens, serve as a migration pathway for leukocytes allowing active immune surveillance (26). CAFs are further characterized based on distinct cellular sources: vascular CAFs (vCAFs), matrix CAFs (mCAFs), cycling CAFs (cCAFs) and developmental CAFs (dCAFs). vCAFs originate from perivascular areas, mCAFs and dCAFs are the product of resident fibroblasts found in TME of genetically engineered MMTV-PyMT mouse model of breast cancer (27). Dominguez and co-workers found TGFβ-driven LRRC15+ CAF lineage in genetically engineered pancreatic ductal adenocarcinoma (28). CAFs are also found to be immunomodulatory expressing MHCII genes and induces antigen-specific ligation with CD4+ T helper cells by showing CD74 in PDAC and named as “antigen-presenting CAFs (apCAFs)” in human PDAC (29).CAFs in late or metastatic tumors differ from early-stage tumors (phenotype diversification presented in **Figure 1**) in having high metabolic synthesis and dysregulated transcriptional profiling. Among the various known functions, CAFs produce ECM components, mediate collagen crosslinking increasing stiffness, and direct cancer cells survival signals. They immunomodulate the TME evading tumor surveillance (22). **Table 1** describes the CAF subtypes and related gene signatures found in TME.

**Table 1:** Cancer-associated fibroblast subtypes found in tumor tissues as revealed by single cell RNA sequencing.

Cancer type/models	CAF subtypes	Markers/gene signature	Significance	References
Lung adenocarcinoma	Matrix fibroblasts	*COL13A1+, COL14A1+*	Present majorly in early-stage tumor tissues	(30, 31)
	Fibroblastic reticular cells	*PDPN, PDGFRA*	Immunologically specialized myofibroblasts that gather immune cells into the lymph node, support T cell, and B cell survival but prevent their activation, maintains dendritic cell migration	
PDAC (Human+Mouse)	Myofibroblasts	*α-SMA++, TAGLN, MYL9, TPM1, TPM2, MMP11, POSTN, HOPX* (contractile proteins)	Found primarily adjacent to the cancer cells, Smooth muscle contraction, focal adhesion, ECM organization, collagen formation	(32)
	Inflammatory CAFs (iCAFs)	*CLEC3B, PDGFRα, CFD, LMNA, DPT, AGTR1, HAS1, HAS2* (matrix proteins) IL6, IL8, chemokines CXCL1, CXCL2, CCL2, and CXCL12	Found in desmoplastic areas away from tumors, activate inflammatory pathways such as IFNγ response, TNF/NF-κB, IL2/STAT5, IL6/JAK/STAT3, and the complement pathway	
PDAC (Human)	Antigen-presenting CAFs (apCAFs)	*COL1A1, COL1A2, DCN, PDPN, MHCII genes (HLA-DRA, HLA-DPA1, and HLA-DQA), CD74*	Have immunomodulatory capacity (activate CD4+ T cells), involved in antigen presentation and processing, fatty-acid metabolism, MYC targets, and MTORC1 signaling	(28, 29)
	Lipofibroblasts	*FABP4, CAR3*	Lipid droplet–containing fibroblasts, express lipid metabolism genes	
Breast Cancer	CAF-S1	*CD29^+^ FAP^++^ FSP1^+^ αSMA^++^PDGFRβ^+^ CAV1^low^*	Key player in immunosuppression, promote differentiation of CD4+CD25+ T lymphocytes into CD25+FOXP3+ cells, characterized by cell adhesion, ECM organization, and immune response	(33)
	CAF-S2	*CD29^low^ FAP^-^ FSP1* ^−^ *^/low^αSMA^-^ PDGFRβ^-^ CAV1* ^−^	Mainly present in LumA tumors	
	CAF-S3	*CD29^+^ FAP^-^ FSP1^++^αSMA^-/low^ PDGFRβ^+^ CAV1^-/low^*	Detected mainly in juxta-tumors	
	CAF-S4	*CD29^++^ FAP^-^ FSP1^+^ αSMA^++^ PDGFRβ^+^ CAV1^low^*	Activated CAF subset, detected in all tumor types LumA, TNBC, HER2, characterized by muscle contraction, regulation of actin cytoskeleton, and oxidative metabolism	
Breast Cancer (MMTV-PyMTmouse model)	Vascular CAFs (vCAFs)	vascular regulatory genes (*NOTCH3, EPAS1, COL18A1, NR2F2*), desmin, Nidogen-2	Originated from perivascular location, significant role in vascular development and angiogenesis	(27)
	Matrix CAFs (mCAFs)	ECM-related genes (*DCN, LUM, AND VCAN*), structural proteins (*COL14A1*), matricellular proteins (*FBLN1, FBLN2,SMOC*), and matrix-modifying enzymes (*LOX, LOXL1*), CXCL14	Found mainly in normal tissues or early tumors, Resident fibroblasts, involved in ECM production, regulates tumor immune response	
	Cycling CAFs (cCAFs)	Similar to vCAFs but have differentially expressed cell cycle genes	Majority cells were in G2, M or S phase of cell cycle unlike others which are in G1 phase	
	Developmental CAFs (dCAFs)	stem cell genes *(SCRG1, SOX9, AND SOX10)*,	Originated from malignant cells undergone EMT, involved in differentiation, development, and morphogenesis	
Breast and lung cancer	CD10^+^GPR77^+^ CAFs	*CD10, GPR77*	Chemoresistance, poor survival	(34)
PDAC	LRRC15^+^ CAFs	leucine-rich repeat-containing 15 (*LRRC15*), *PDPN*	TGF-β driven cell population, high expression is associated with poor anti-PD-L1 therapy response	(28)

### Non-Coding RNAs Involved in Fibroblast Differentiation to CAFs and Its Subtypes

Despite being highly researched, CAFs are still a mystery and can be a great tumor-targeting cell if explored. It is a dynamic population in TME that is pluripotent and is known for differentiating into other cell types. Various reviews have highlighted the miRNAs involved in fibroblast differentiation, tumor progression, and metastasis (12, 22). In breast, ovarian, and endometrial cancers downregulation of miR-31, miR-214, miR-148a, miR-205, miR-200b, miR-200c, miR-141, miR-101, miR-342-3p, let-7g, miR-26b, miR-15a, miR-16, and upregulation of miR-155, miR-221-5p, miR-221-3p miRNAs have shown significant regulation in the tumor microenvironment. Reversal of these microRNAs reversed the CAFs’ phenotypic and genotypic characteristics (35). Recently identified non-coding RNAs miR877-3p, and miR-133a targets TGF-β axis causing myofibroblast differentiation (36, 37). Twist1 induced miR-199a-3p suppress caveolin-2 and activates myofibroblast differentiation *via* TGF-β pathway (38). LncRNAs further plays a significant role in determining fibroblast subtypes. LINC00092 long non-coding RNA upregulation is co-related with CXCL14-mediated CAF progression in ovarian cancer cells. LINC00092 overexpression is related to poor clinical prognosis and increased glycolysis leading to metastasis (39). Tong and co-workers discovered the role of exosomal lncRNA POU3F3 in esophageal squamous cell carcinoma (ESCC). POU3F3 regulates fibroblast differentiation to CAFs and causes cisplatin resistance in ESCC. Many lncRNAs with similar roles in different cancers are known: CASC9, PART1, CCAT1, TTN-AS1, DNM3OS, FMR1-AS1, LINC01419, NMR, PCAT1, ROR (40). Aberrant circRNA expression is co-related with myofibroblast differentiation and tumorigenesis. circHIPK3 regulates lung fibroblast-to-myofibroblast transition by functioning as a competing endogenous RNA. circHIPK3 functions as an endogenous miR-338-3p sponge and inhibit miR-338-3p activity, increasing SOX4 and COL1A1 expression and fibroblast differentiation (41). Advances in recent research and use of circRNA databases (for example, MiOncoCirc) are required further to identify the role of circRNA in cellular plasticity variations and using them as therapeutic options.

## Profiling of Phenotypically Abnormal Tumor Endothelial Cells

Endothelial cells are in a constant mode of activation, quiescence, and re-activation, depending on the growing tumors’ metabolic needs and requirements (42). Individual endothelial cells adopt different phenotypes and functions according to the tumor requirements. Endothelial cell phenotypes are primarily divided into tip cells and stalk cells that exhibit distinct genotypes (42). With advances in technologies and the advent of scRNA seq techniques, different subpopulations are discovered. The first parameter to distinguish ECs from the rest of the tumor cells is a separation through a pan-hematopoietic marker (CD45−) combined with CD31, CD144, and vWF. CD31 is a transmembrane glycoprotein that forms the intercellular junctions, CD144 (VE-Cadherin) is an endothelial adhesion molecule and vWF (von Willebrand Factor), a glycoprotein that mediates platelet adhesion in the endothelium (43). All these are preliminary markers to separate endothelial cell population. Other EC identification markers include tip genes (ESM1, NID2, KCNE3, DLL4, RAMP3, EDNRB, CLDN5), capillary markers (CA4, CD36), ACKR1 gene expression by high endothelial venules, arterial (FBLN5, GJA5), lymphatic markers (PROX1, PDPN), pericyte marker RGS5, non-myeloid specific marker AIF1 in different cancer types (44). Stalk endothelial cells generally express VWF, SELP, ACKR, and TMEM252. Several pro-angiogenic factors, metabolic signatures, and transcription factors have differential expression in tumor endothelial cells compared to non-tumor cells like HSFG2 (44). Lambrechts and co-workers classified tumor endothelial cells in different clusters based on the marker genes identified; Lymphatic endothelial cells (PDPN+, PROX1+), tumor-derived blood endothelial cells (FLT1+, IGFBP3+, and SPRY1+), malignant, and non-malignant endothelial cells (23) (**Figure 1**). Transcriptional and epigenetic dysregulation in TME triggers the formation of these angiogenic candidates and their subtypes from healthy blood endothelial cells. TEC subtypes lead to loss of vascular integrity, structure fragile and leaky blood vessels, and migration of immune cells, thus contributing to the growing complexity of tumors (45). Goveia and co-workers extend the endothelial heterogeneity by discovering previously unknown functionally validated endothelial phenotypes across patients and in-vitro/in-vivo models. Non-malignant lung (hpNECs) tissues have a comparatively high abundance of postcapillary, alveolar type II, scavenging, and lymphatics endothelial cells than aggressive tumors. TEC phenotypes were majorly immature ECs, tip cells, patient-specific and lymphatics hTECs. Arterial, activated postcapillary veins, and alveolar type II phenotypes are common in non-tumor and tumor tissues (46). They have also identified top-ranked markers of each phenotype and specified roles in tumor progression (listed in **Table 2**). The top-ranked marker genes have significant roles in regulating immune surveillance, EC migration, matrix remodeling, VEGF signaling, and angiogenesis by increasing growth factors and chemical stimuli that triggers angiogenic cascade within TME, including vascular endothelial growth factor (VEGF), fibroblast growth factor (FGF), platelet-derived growth factor (PDGF), angiopoietins (Ang), hepatocyte growth factor (HGF), hypoxia-inducible factor (HIF), insulin-like growth factor (IGF), transforming growth factor-beta (TGFβ), matrix metalloproteinase (MMP), and tumor necrosis factor (TNF) (54). Blood vessels built of TECs have loose interconnecting tight junctions, irregularly shaped, tortuous with high interstitial pressure, and ill-organized. TEC release pro-angiogenic factors such as VEGF, PDGF-β, FGF, and EGF show chromosomal abnormalities and are resistant to anticancer drugs (55). TEC derived cadherin-2 induces HIF-1α/VEGF mediated angiogenesis by regulating MAPK/ERK and MAPK/JNK signaling pathways (56).

**Table 2:** Molecular profiling of endothelial cell heterogeneity in tumor human/mouse tissue samples.

Endothelial cell phenotype	Transcriptional profiling	Major functions/activated signaling pathways	References
Tip cells	*ESM1, APLN, ADM, MMP14, ANGPTL2, INSR, PDGFB, CXCR4, PGF, KCNE3, DLL4, RAMP3, EDNRB, LXN, CD34*	Cytoskeleton remodeling, involved in VEGF signaling, EC migration, extracellular matrix formation, collagen production	(30, 46, 47)
Stalk cells	*VWF, SELP, ACKR1, SPINT2, VEGFR2*,	Known to originate from tip cells, maintains endothelial cell polarity, lumen formation	(47, 48)
Lymphatic ECs (LECs) and subtypes	*LYVE1, CCL21, TFF3, MMRN1, ADIRF, AKAP12, FABP4, CD9, ANXA2, SNCG, GYPC, S100A10, VIM, PPF1BP1, EFEMP1, PROX1, PDPN*	Secrete SEMA4C promoting metastasis	(46, 49)
	LEC I: *ACKR4, NT5E*	Not known	
	LEC II: *TNFRSF9, MARCO, IFNGR, CSF1, CXCL1-CXCL5*	Express chemokines and cytokines such as IL6, IL33, IFNGR1, IFNGR2, role in macrophage homeostasis, express neutrophil chemoattractants	
	LEC III : *ACKR4, NT5E, LYVE1, MFAP4*	Not known	
	LEC IV : *PDPN, LYVE1,CCL21*	Not known	
	LEC V:*CLDN11*	Not known	
	LEC VI : *MARCO, LYVE1, CSF1, CXCL1-CXCL5*	Express chemokines and cytokines such as IL6, IL33, IFNGR1, IFNGR2, role in macrophage homeostasis, express neutrophil chemoattractants	
Endothelial progenitor cells	*TIE2, FGF3, VEGRF2, CD34*	Promote vascular regeneration and repair	(50)
Tumor ECs	VEGF	Notch signaling	(46)
Arterial ECs	*GJA4, GJA5, SOX17, EFNB2, DKK2, CXCL12, FBLN5, JAM2, FBLN5, CYTL1*	Involved in vascular integrity, homeostasis, and vasotonus	(46, 51)
Type I alveolar capillaries	*EMCM, VWF ^(low)^, podoplanin, aquaporin5*	Anti-microbial defense	(46)
Type II alveolar capillaries	*FCN3, SLC6A4, TMEM100, IL7R, BTNL9, TNFSF10, VIPR1, CD14, NOSTRIN, HIF3A, VWF*	Vasoregulation	(46)
Postcapillary veins	*VWF1, ACKR1, SELP, VCAM1*	Leukocyte recruitment, regulation of blood pressure and perfusion	(46, 52)
Large vein ECs	*VWF, CYTL1, PI16, NOS3, LCN2, SLC38A5*	Immunomodulatory functions	(46, 51)
Post capillary vein ECs	*ACKR1, SELP, VCAM1, CCL14, POSTN, ACTN1, PRCP, NPC2, DUSP23*	Leukocyte recruitment, tissue perfusion, and pulmonary blood pressure, involved in immune cell recruitment	(46)
Capillary ECs	*MHC-II, MFSD2A, RGCC*	Involved in antigen presentation	(46, 51)
Scavenging capillary	*AIF1, APOC1, APOE, CCL18, CD36, CD52, CD68, CD74, CRIP1, CYBA, FCER1G, FCGR3A, FTH1, FTL, LST1, LYZ, MARCO, MNDA, MSR1, OLR1, TREM1, TYROBP*	Adhesion, antigen presentation, pathogen clearance, inhibition of tumor angiogenesis	(46)
Proliferating ECs	*TOP2A, MKI67, CCDC34, CDKN1A, CENPA, CKS1B, CKS2, EZH2, FEN1, H2AFZ, HMGB2, HMGN2, HSP90AA1, LGALS1, LIG1, LMNB1, NRM, PCNA, RRM1, SMC2, SPC24, STMN1, TUBA1B, TYMS*	Basement-membrane breaching, immune cell recruitment, and semi-professional antigen presentation, tumor angiogenesis	(46, 51)
Breaching ECs	Tip cell markers (*DLL4, CXCR4, APLN*), collagen remodeling markers (*COL4A1, COL4A2, COL18A*), *HSPG2, IGFBP3, LAMB1, MCAM, PXDN, LAMA4, NID2*	Upregulates expression of tip cell markers, initiation of vessel sprouting, involved in collagen remodeling and basement membrane degradation	(46)
Immature ECs	*ID2, ID3, ENG, PLVAP, GSPG2, APLNR, VWA1, RBP7, GSN, MMP2*, VEGF receptors (*KDR, FLT1, TIE1*)	Notch signaling, develop into tumor ECs	(46)
Capillary-arterial ECs	*TGFB2, GLUL*	Express both capillary and arterial markers, VEGFA, Notch signaling	(46, 53)
Capillary-venous ECs	*TFRC, CAR4*	Express both capillary and venous markers, VEGFA, Notch signaling	(46, 53)

### Non-coding RNAs Regulation in Tumor Angiogenesis

Controlling vascular inflammation and angiogenesis is a limiting step to control tumor metastasis. Activation of tumor endothelial cells involves a cascade of events involving regulation by non-coding RNAs. They regulate transcriptional factors and governance mechanisms of neighboring genes. Various miRNAs are long known to regulate angiogenesis in tumor microenvironment. A comprehensive overview of pro-angiogenic and anti-angiogenic miRNAs is given in **Table 3**. lncRNA acts as molecular decoys of RNA binding proteins and are known to regulate protein-coding genes *via* interaction with transcriptional factors and other binding proteins (65). MVIH was the first lncRNA known that accounts for tumor angiogenesis in hepatocellular carcinoma (66). lncRNA regulate angiogenesis by activating oncogenic signaling pathways like NF-κβ, STAT-3, AKT, mTOR, WNT. Man and co-workers reported a set of endothelial cells enriched with lncRNAs and identified the role of spliced-transcript endothelial-enriched lncRNA (STEEL) in angiogenic potential, macrovascular/microvascular identity, and shear stress responsiveness (67). STEEL upregulates eNOS and transcription factor Kruppel-like factor 2 (KLF2) in endothelial cells. Some of the other known lncRNAs include MALAT1, MANTIS, PUNISHER, MEG3, MIAT, SENCR, GATA6-AS, WTAPP1, CCDST, PVT1, CamK-A, UBE2CP3, HULC, OR3A4, LINC01410 each having specific roles in tumor angiogenesis (68). Wang and co-workers recently discovered a lncRNA named HITT (HIF-1α inhibitor at translation level) that is down-regulated in multiple human cancers (69). In hypoxic conditions, miR-205 upregulation causes HITT degradation and that allows YB-1 (Y-box binding protein) translational regulator binding on 5′UTR HIF-1α mRNA region leading to tumor angiogenesis. circRNA is another group of lncRNAs regulating angiogenesis (69). Multiple circRNAs acts as miRNA sponges and regulate the mRNA expression of targeted angiogenic and vascular sequences. Yang and co-workers identified an inhibitory role of forkhead DNA-binding protein-Foxo3 circRNA on tumor growth and angiogenesis. circFoxo3 binds to miR-22, miR-136, miR-138, miR-149, miR-433, miR-762, miR-3614–5p, miR-3622b–5p and promotes translation of Foxo3 mRNA, which is a tumor suppressor gene (70). Similarly, circ_0003575, circ_0003204, circ_002136, and circ-001971/miR-29c-3p plays an important role in angiogenesis (71–73),circ_001621/miR-578 regulates VEGF/HIF-1α axis controlling breast cancer angiogenesis (74), circ_0007059/miR-378 regulates EMT transition in lung cancer cells (75). Also, long intergenic non-coding RNA-p21 (lincRNA-p21) is regulated by TP53 and angiogenesis-related genes (76).

**Table 3:** List of miRNAs regulating angiogenesis in tumor microenvironment.

	miRNAs	Targeted signaling pathways	References
**Proangiogenic**	miR-9, miR-21, miR-874, miR-16, miR-34a, miR-590-5p, miR-132, miR-296, miR-210, miR-568, miR-25-3p, miR-150, miR-499	VEGF signaling	(57–59)
	miR-210, miR-155, miR-21, miR-200c, miR-199	HIF signaling	(57, 58, 60, 61)
	miR-375, miR-296, miR-146a	PDGF	(57, 62)
	miR126, miR-382, miR-26a, miR-145	RTK signaling	(57, 62)
	miR-9, miR-26-3p, miR-146a-5p, miR-98, miR-181a-5p	MMP signaling	(57, 62)
	miR-503	FGF signaling	(57, 62)
	miR-204	Angiopoietin	(60, 61)
	miR-494	Akt/eNOS pathway	(60, 61)
	miR-93	Integrin-β8	(63)
**Anti-angiogenic**	miR-15a, miR-16, miR-29b, miR-29c, miR-128, miR-497, miR-126, miR-503, miR-204, miR-195, miR-124, miR138, miR-134, miR-107, miR-206, miR-622	VEGF signaling	(57, 59, 61, 62)
	miR-519c, miR-22, miR-107, miR-204, miR-128, miR-145, miR-206	HIF signaling	(57, 61, 62)
	miR-125b	VE-cadherin	(57, 62)
	miR-29b, miR-204, miR-200c	PDGF	(57, 62)
	miR-21, miR-218, miR-18a, miR-145	RTK signaling	(60, 61)
	miR-29b	MMP signaling	(57, 62)
	miR-503, miR-148b-3p	FGF signaling	(57, 62)
	miR-542-3p, miR-543	Angiopoietin	(57, 62)
	miR-206	Akt/eNOS pathway	(60, 61)
**Pro/Anti-angiogenic**	miR-27b	VEGF, AMPK, Semaphorin 6A	(57, 62, 64)
	miR-17-92	HIF-1α, VEGF	
	miR-19a	MMP9, VEGF	

## Immune Landscape

Immune cells are prime in TME that initially guards the body but eventually turns into a tumor supporting population (10). Both myeloid and lymphoid lineage have pro-tumoral and anti-tumoral roles depending upon the stage of cancer. For instance, macrophages promote T cells’ activation to clear tumor cells at early stages but prevent T cells from even recognizing the tumor cells as a tumor progresses (41). Immune cells communicate among each other and regulate mechanisms linked to tissue homeostasis and altered patient survival. Secretions from immune cells also shape the activity within TME. The secretion of CCL5 and XCL1 from NK cells attract antigen-presenting dendritic cells. IFNγ secretion promotes macrophage polarization and Th1 cell activation that triggers the immune microenvironment against tumor cells (77). In response, tumor cells secrete factors such as pro-inflammatory cytokines like IL8 and CXCL-1, 2, 8 that attract neutrophils. Neutrophils produce neutrophil extracellular traps (NETs) that shield the tumor cells from cytotoxic CD8+ T cells, NK cells, and minimize the effect of immunotherapies (78). Therefore, growing tumors are continuously combating the body’s immunity, which they eventually defeat. Immune cells are highly complex and consist of several distinct lineages that make them hard to study and target (79). Recognizing each immune cell’s indispensable role in supporting tumor proliferation, it would help us curb immunosuppressive reactions and promote immuno-stimulatory functions. scRNA-seq is a high-throughput approach that analyses the immune cells that typically display diverse phenotypes in-vivo (80). It identifies transcriptomics at single cell level by applying data processing pipelines including demultiplexing, sequence QC, alignment, and transcript quantification (81).

## Monocytes and Macrophages Are Phenotypic Markers of Aggressive Tumors

**Figure 2:**
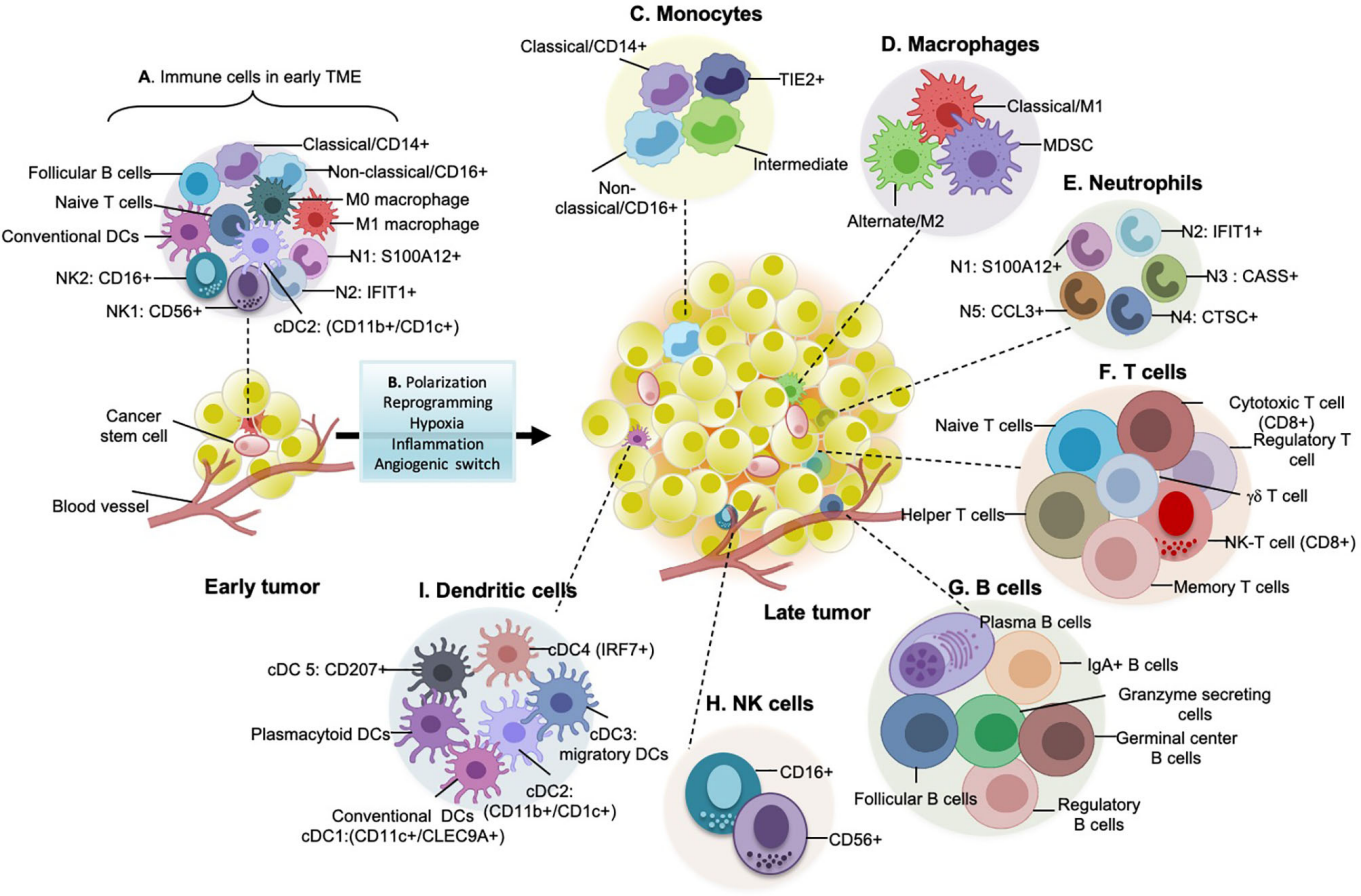
Immune landscape in early and late tumors. **(A)** Early tumors consist of non-aggressive hemopoietic cells such as classical monocytes, M0 macrophages, M1 pro-inflammatory/anti-tumor macrophages, NK1 and NK2 natural killer cell subtypes, conventional dendritic cells, naïve T cells, Follicular B cells. **(B)** As the tumor progresses to advanced stages, multiple immune cells converge to support pro-tumorigenic/anti-tumorigenic functions. Tumor-derived factors such as TGF-b, FGF or PDGF, interleukins, etc. are responsible for the diversification of immune cell types during cancer development. Moreover, with an increase in hypoxic and inflammatory core sites and activation of angiogenic switches, immune cells alter their conventional anti-tumorigenic behavior to pro-tumorigenic potential. **(C)** Monocyte subtypes, especially classical CD14+, Non-classical CD16+, TIE2+ cells, and intermediate cells, are present in late tumors. Hypoxia is a well-defined factor that leads to the regulation and infiltration of these monocytes into TME. **(D)** Types of macrophages present in late tumors: M2 (pro-tumorigenic) is the massively distributed subtype in late tumors and is related to poor prognosis and patient survival. They secrete a plethora of pro-tumorigenic proteases, cytokines and growth factors (for example, VEGF, EGF, which participates in a paracrine signaling loop through tumor-secreted CSF-1). The number of M1 macrophages are less in late tumors but comparatively higher in early tumors as they are anti-tumorigenic. MDSCs (myeloid-derived suppressor cells) are the recently discovered heterogeneous group of immune cells that are immunosuppressive. **(E)** Types of different neutrophils present in late tumors: majorly 5 types of neutrophils are known which are evolutionarily categorized from two subsets i.e., pro-tumorigenic and anti-tumorigenic. **(F)** Subtypes of T cells that play a role in shaping tumor immunity: helper, cytotoxic and effector memory T cells fight against growing tumors directly or indirectly by antigen presentation, cytotoxic granzyme and perforin release, cytotoxic T cells upon exhaustion are termed as exhaustive T cells and are signature of aggressive tumors, regulatory T cells are immunosuppressive, therefore linked with poor patient survival, gdT cells are a significant focus of cancer immunotherapy as they have strong cytokine production ability. **(G)** Types of B cells found in late-stage tumors, their antibody-secreting skills are linked with prolonged patient survival. But regulatory B cells are immunosuppressive. **(H)** Natural killer (NK) cells shaping tumor immunity: these are the cytotoxic cells and express a specialized class of natural cytotoxicity receptors (NCRs) such as NKp30, NKp44, and NKp46 which initiate tumor targeting by recognition of heparan sulphate on cancer cells. **(I)** Different types of dendritic cells (DCs) present in late-stage tumors: these are the active cells and express co-stimulatory molecules having efficient anti-tumor immune responses.

Monocytes/macrophages are the major components of the tumor ecosystem (23). Human monocytes are divided into 3 largest clusters: classical (CD14++, CD16−), intermediate (CD14+ CD16+), and non-classical (CD14+ CD16++) (82). Recent mass cytometry (CyTOF) and single-cell studies have identified new monocytic markers in tumor cells: CD68, CSF1-R, CSF2-R, CD11C, CD1C, CD141, and HLA-DR surface markers (83). A subset of monocytes expressing angiopoietin receptor TIE2 play a significant role in tumor angiogenesis. TIE2 monocytes are CD54+ CD11b+ CD16+ CD14^low^ L-selectin-CCR2− and its expression is upregulated in response to hypoxia (84). Monocytes uniquely differentiate into immunosuppressive macrophages rather than into immunostimulatory DCs stimulated by the tumor secretions (85). Macrophages differ from monocytes by having a unique set of markers (D68, MSR1, MRC1) (44). Monocytes are recruited to the TME by chemoattractants like CCL2, CCL4 which further differentiate into TAMs. Conventionally, macrophages are divided into two major groups CD14+ S100A8/9+ M1-like (classical macrophages) having anti-tumor functions and CD16+(FCGR3A) M2-like (alternate macrophages) with pro-tumorigenic phenotypes (86). The percentage of TAM infiltration is a marker of aggressive disease and poor prognosis (87). Thorsson and colleagues characterized the dominance of monocytes/M2 macrophages associated with the worst prognosis while evaluating the immunogenomic signature in around 33 cancer types (88). M2 macrophages shows aggressive tumor phenotype associated with tumor growth, angiogenesis, immune evasion and cancer stemness. It also support mutagenic microenvironment and cancer initiation by producing pro-inflammatory cytokines (IL6, TNF-α, IFN-γ), growth factors (VEGF, EGF), proteases and reactive oxygen species (30). Tumor tissues are enriched in recruited macrophages known as mo-Macs that are ontogenically different from resident macrophages and are both anti and pro-inflammatory (30). The macrophage population varies spatially depending upon its location in growing tumors (tumor and juxta-tumoral regions), and tissue specificity. Tumor-specific subtypes as revealed by single-cell technology include early immigrant macrophages (HLA-DR+CD192+), monocyte resembling immature macrophages (CCR2+), tissue-resident macrophages (CD206+HLA-DR+), TAMs (CD64+HLA-DR+), and myeloid-derived suppressor cells (MDSC; HLA-DR−/low) (19, 44) (**Figure 2**). Quin and co-workers precisely identified the types of M2 type macrophages in heterogeneous tumor microenvironment based on gene expression: PPARG+ macrophages exclusive to normal lung tissue, CCL18+ macrophages cluster and GPNMB, MMP9+ cluster having a role on tumor remodeling, CX3CR1+ cluster involved in pathogen and apoptotic cell clearance (44). The recent subtype identification by Qian and colleagues demonstrated the role of macrophages in tumor progression, promoting angiogenesis and invasion, and suppressing T cell responses (44). Many reports have also indicated the presence of PD-L1 in pro and anti-inflammatory markers (19, 89). PD-L1 TAMs are phenotypically heterogeneous and express CD38, pro-tumor markers CD204, CD206, CD169, and CD163, and the anti-tumor marker CD169 (19). Some monocyte and macrophage subsets are mentioned in **Table 3**.

### Regulation of TAM Phenotype by Non-Coding RNAs

TAMs association with immunosuppressive and tumor supporting M2 phenotype necessitates TAM reprogramming that would act inversely and help cure tumors. The potential ability of TAM reprogramming towards antitumor phenotypes largely depends upon transcriptional control. There are a set of miRNAs involved in alternative macrophage activation: miR-9, miR-21, miR-375, miR-340-5p, miR-125a, miR-511, miR-92a, miR146a, miR-147, miR-145-5p, miR155, miR-187 (90, 91). The functional and biological relevance of lncRNAs is in the initial phase of research. RP11-361F15.2 is a protumoral lncRNA that promotes M2-like polarization of TAMs by inversely regulating miR-30c-5p and plays a vital role in cancer invasion (92).

Similarly, BCRT1 is an M2 polarizing lncRNA that is upregulated in response to tumor hypoxia and leads to breast cancer progression *via* novel HIF-1α/lncRNA BCRT1/miR-1303/PTBP3 pathway (93). LncRNA RPPH1 mediates protumoral macrophage polarization by upregulating anti-inflammatory cytokines and also leads to colorectal cancer migration and metastasis by preventing β-III tubulin (TUBB3) ubiquitination (94). Many lncRNAs are known (NEAT1, TUC339, MM2P, MALAT1, ANCR), and many are still in research (95–97). CircRNAs also have regulatory functions but, not much is known about the role of circRNA in macrophage polarization as it relatively new field and holds promises in future therapeutics. Such discoveries would help us to understand precision medicine, some can be a biomarker depending upon its abundance in a particular tumor, and some can be a great therapeutic tool.

## T Cell Interactome Within TME

The complexity of tumor-infiltrating T cells suggests a strong influence of tumors on the T cell transcriptome. T cell population is generally sorted from the rest of the immune cells by CD3+CD4+CD8+CD25+ cell surface markers (98). They are classified conventionally as Naïve, effector, and memory T cells. In a specific study by Lambrechts and co-workers on NSCLC, single-cell sequencing reveals T cells clusters into regulatory T cells (FOXP3+), natural killer and natural killer T cells (FGFBP2+), CD8+ T cells (CD8+, naïve, effector, resident memory or exhausted), CD4+ T cells (CD4+), and minor populations of γδ T cells (23) (**Figure 2**). Following infiltration, naïve T cells differentiate into effector T cells that further activate into cytotoxic or central memory T cells (99). Primary tumors are enriched mainly with effector T cell subtypes characterized by high expression of chemokine receptors and cytotoxic genes like CD28, ICOS, OX40, CD40L, and CD137 and show low expression of exhausted T cell population (89). With tumor progression from primary to metastatic sites, expression of co-inhibitory receptors CTLA-4, PD-1, TIGIT, LAG3, TIM-3, and CD160 leads to progressive T cell dysfunctioning (89). Percentage of programmed cell death protein 1 (PD-1+), mucin domain protein 3 (TIM-3+), and cytotoxic T lymphocyte antigen-4 (CTLA-4+) cytotoxic and regulatory T cells increases in juxta-tumoral tissues. Cells expressing these co-inhibitory receptors are immunosuppressive, they originate from various sources influenced by TME; by migration from circulatory systems, effector T cell conversion, differentiation resulting from the suppression of APCs, etc (100). These suppress the NK cell activity, cytotoxic T cell response; thus, performing pro-tumoral functions (100). According to Qian and colleagues, some of the T cell clusters express NK cell markers (KLRD1, FGFBP2, CX3CR1), suggesting they are endowed with NK T-cell (NKT) activity (44). Currently, known T cell subtypes, marker profiling, and associated roles in TME are highlighted in **Table 3**.

### Regulation of T-Cell Function by Non-Coding RNAs

T cells are at an extremely exhaustive stage in advanced tumors and require regulatory or transcriptional boosting to fight tumors. Non-coding RNAs regulating the T cell differentiation functions, harnessing T cell survival activities would be beneficial as therapies. miR-155 epigenetically silences the CD8+ T cell differentiation by enhancing Polycomb repressor complex 2 (PRC2) activity that blocks the transcription factors (*EOMES, ID2, PRDM1, ZEB2, MAF, NR4A2*) which are known to drive terminal differentiation and exhaustion (101). miR-28 is down-regulated in around 30% exhaustive T cell population. It can be an important therapeutic target as it inhibits the expression of the immune checkpoint molecules PD-1, TIM3 thus preventing exhaustive T cell differentiation (102). miR-16, miR-21, miR-142-3p, miR-142-5p, miR-150, miR-15b, miR-181a, and let-7f miRNAs are over-expressed in naïve T cells, and their expression reduces significantly in effector T cells (103, 104). Furthermore, miR-17-92, miR-21, miR-155, and miR-132-3p are over-expressed in effector and memory T cells compared to naïve T cells (103). Min and colleagues searched for miRNAs involved in DC differentiation to Treg cells and found the novel role of miR-27a in T cell differentiation. miR-27a is known to hamper Th1 and Th17 cell differentiation and help in the DC-mediated Treg (CD4+CD25+Foxp3+) population differentiation *via* pro-inflammatory cytokine production (105). Hence it is a potential target for cancer immunotherapy. Xu and co-workers demonstrate the role of miR-424 (322) in regulating the PD-L1/PD-1 and CD80/CTLA-4 axis in chemo-resistant ovarian cancer. miR-424 (322) restoration leads to PD-L1 blockade and activation of CD8+ T cells, thus preventing chemo-resistance inversely correlated with PDL1, PD1, CTLA-4 expression (106). The role of lncRNAs and circRNAs in T cell function is less explored area of research. Kotzin and colleagues discovered the role of lncRNA Morrbid in CD8+ T cell differentiation in response to IFNγ viral infection (107). This lncRNA may have some role in tumor T cells as well but needs confirmation. Wang and co-workers discovered circRNA-002178 acting as a “competing endogenous RNA” (ceRNA) to promote PDL1/PD1 expression in lung adenocarcinoma. circRNA-002178 could enhance PDL1 expression *via* sponging miR-34 in cancer cells to induce T-cell exhaustion (108). Thus circRNA-002178 reversal therapies may work against tumor supporting T cell population.

## B Cell Transcriptome in TME

B cells are adaptive immune cells associated with prolonged patient survival (109). They are infiltered within TME *via* CXCL13 secretions or antigens from tumor cells (110). B cells are relatively abundant in tumor samples compared to non-tumor (30) and are comparatively sparse compared to T cells in TME (111). B cells have five differentiated states: follicular B cells expressing CD20 (MS4A1), CXCR4, HLA-DRs, plasma B cells expressing immunoglobulin gamma (IgG) (MZB1 and SDC1/CD138), mucosa-associated lymphoid tissue-derived plasma B cells expressing IgA (CD38+), granzyme B-secreting B cells, and germinal center (GC) B cells (23). Individually, follicular B cells include mature-naïve (IGHM, CD72, CD27−) B cells that gives rise to memory B cells (IGHG1 and CD27+) while migrating through the germinal center (GC) (30). Some B cells have CD27− memory B-cell phenotype and express antigens associated with antigen-presenting cells (APCs) (namely, MHC class II, CD40, CD80, and CD86) (112). B cells residing in TME are exhaustive compared to ones in the non-tumorigenic environment as indicated by reduced protein secretion, and impairment of Myc and mTOR pathways (23). Mature-naïve B cells differentiate to IgM+ GC memory cells that further produce IgM- cells by undergoing a class-switch recombination mechanism. These IgM+ and IgM- cells finally produce GC dependent memory B cells or plasma cells. IgM- memory B cells differentiate into either IgG+ or IgA+-expressing plasma cells (44) (**Figure 2**). Takemori and co-workers also identified a distinct class of memory B cells that are not matured in GCs but are a response to T cell antigens (113). Expression markers of B cell subtypes are mentioned in **Table 3**. B cells promote antitumor immunity by driving antibody-dependent cellular cytotoxicity (ADCC), phagocytosis, complement activation, T cell activation, stimulating cytotoxic immune responses, and releasing granzyme B and TRAIL factors (114). B cell also have immunosuppressive pro-tumorigenic subsets such as regulatory B cell (CD1d+CD5+CD19+, CD5+CD19+) and CD5+ B cells (115). They are known to modulate the secretion of immunomodulatory cytokines such as IL10 and TGFβ, activate STAT3 functioning, and stimulates Treg formation (116).

### B Cell Development and Activation by NON-CODING RNAs

miRNAs have a significant role in B cell development, activation, malignant transformation, and functions. There are some well-known miRNAs regulating B cell differentiation, proliferation and activation: miR15a, miR21, mir29, miR-17/92 cluster, miR-23a, miR-34a, miR-142, miR-150, miR-155, miR-181 family, miR-181a1b1, miR-212/132 cluster, miR-9, miR-17/92 cluster, miR-30, miR-125b, miR-155, miR-181b, miR-210, miR-221, miR-223, miR-148a, miR-146a (117, 118).miRNA 15A and 16-1 activate signaling pathways that mediate chemotaxis of immune regulatory B cells to colorectal tumors (119). lncRNAs are classified into six functional groups based on the functional chromatin features: eRNAs, promoter lncRNAs, bivalent lncRNAs, repressive lncRNAs, CTCF lncRNAs, and other lncRNAs (120). Zhou and co-workers discovered five B-cell-specific lncRNAs computationally (TNRC6C-AS1, WASIR2, GUSBP11, OGFRP1, and AC090515.2) associated with improved prognosis, and three B cell specific lncRNAs (PART1, MAFG-DT, and LINC01184) associated with poor prognosis in bladder cancer (121). Pyfrom and colleagues studied B cell-specific lncRNA BCALM (AC099524.1) highly expressed in various cancers. AC099524.1 is necessary for the interaction of signal transduction proteins PLD1 and AKAP9 and have implications in B cell immune response (122). Various other lncRNAs are known to have a role in B cell development: LEF-AS1, MYB-AS1, CRNDE, and SMAS-AS1 (123). The role of circRNA in tumor B cell activation is not yet explored.

## NK Cells and Other Dendritic Cells

Natural killer cells belong to an innate lymphoid family having cytotoxic and cytokine-producing potential and can identify tumor cells with the help of a unique set of receptors. NK cells are distinct from the rest of the immune cell population by having distinct cell surface markers CD3−/CD56+ or CD3−CD16+. They are majorly divided into different subpopulations depending upon CD16 and CD56 markers’ expression, each with distinct phenotypic properties (124). The presence of NK cells in tumors depends upon the stages and the cancer types. Tumor-specific NK cells exhibits high expression of CD69 and NKp44 activation markers and low expression of NKp30, NKp80, DNAM-1, CD16, and ILT2 as compared to peripheral blood and normal lung NK cells (125). Some NK cells strongly correlate with T cells and are popularly known as Natural Killer T (NK-T) cells (**Figure 2**). Dendritic cells are of multiple specialized subtypes (mentioned in **Table 4**) present in TME that are important for antigen presentation, phagocytosis, and adaptive immune responses. The historical origin of dendritic cells is through lymphoid lineage. The differentiating markers of DCs from the rest of the immune cells are HLADR+ lineage– cells. Previously known DC subtypes include CD11C+ (Itgax) conventional DCs (cDCs) that are further characterized into either CD141+ or CD1C+ cells, and CD123+ (Bst2, Siglech) plasmacytoid DCs (pDCs) (82). Qian and colleagues identified five different DC subtypes in heterogenous TME including conventional DCs (cDC1: CLEC9A, XCR1, BAFT3; cDC2: CD11b, CD1C, CLEC10A, SIRPA; cDC3 which include migratory DCs and expresses CCR7, CCL17, CCL19 gene expression, cDC4: pDCs expressing LILRA4, CXCR3, IRF7, and cDC5 expressing CD207 and Langerhans cell-specific markers) (44). Davidson and co-workers further classified dendritic cells based on their presence in tumors or lymph nodes (LN). Tumor cDC1 clusters express the dermal DC marker Cd103 (Itgae), whereas the LN population expresses CD8a marker specific to LN resident dendritic populations (18). Transcriptionally, tumor DCs are more active and display a wider expression of different co-stimulatory molecules but lack T cell provoking stimulations than their counterparts.

**Table 4:** Distinct subsets of immune cells in human/mouse tumor tissues.

Myeloid lineage						
Cell type	Subtypes		Expression markers		Significance/Functions	References
			Human	Mouse		
**Monocytes**	Classical		*CD14++, CD16*−*, FCN1*	*Ly6C+ CCR2+CX3CR1^int^*	Phagocytosis, innate sensing/immune responses and migration	(56, 79, 83, 84)
	Non-classical		*CD14+, CD16++, CDKN1C, LILRB2, ITGAL*	*Ly6C^low^ CCR2*−*CX3CR1++*	Present antigen processing capabilities, promote neutrophil adhesion at the endothelial interface *via* the secretion of TNF-α	
	Intermediate		*CD14++, CD16+, S100A8, S100A9,CSF3R, CCR5*	*S100A8, S100A9, CSF3R, CCR5*	Express antigen presentation-related molecules, secrete TNF-α, IL-1β, IL-6, and CCL3 upon TLR stimulation	
	MonoDC		*CLEC10A*, MHC class II genes*,CD74*		Not known	
	TEMs		*Tie2/TEK* angiopoietin receptors		Tumor angiogenesis	
	Classical (M1)		*CD14+ S100A8/9+*		proinflammatory/antitumorigenic, polarize to M2 type in advance stage cancers	
	Alternate/anti-inflammatory/protumorigenic (M2)		*CD16+(FCGR3A), CD204, CD206,CD163*		promote immunosuppression, supportformation of abnormal vessels in TME that lead to tumor progression	
	Myeloid-derived suppressor cells (MDSC)		*HLA-DR^-/low^*		Immunosuppressive activities, promote tumor cell survival, angiogenesis	
**Dendritic cells**	DC1		*CD141+, XCR1, CLEC9A, CADM1*		Antigen presenters to CD8+ T cells	(79, 82)
	DC2		*CD1C+, CD1A,CD1C, CD1E, CD207, FCER1A*		Interact with CD4+ T cells	
	DC3		*CD1C-, CD141*−*, FSCN1, BATF3, IRF8, CCR7, LY75, CCL22, BIRC3, NFKB2, IL12B*		Activated DC phenotype	
	Plasmacytoid DCs (pDC)		*TCF4, LILRA4, CLEC4C (CD303), IL3RA*		Initiate inflammatory responses, produce IFN-γ, also known to promote immunosuppressive environment	
**Neutrophils**	Human	Mouse	Human	Mouse		(79, 126)
	N1	N1	*S100A12*	*MMP8*	antitumor activity, induce activation of T, B, NK and DC cells, ROS production	
	N2	N2	*IFIT1*	*IFIT1*	pro-tumor activity, secrete ECM remodeling enzymes and pro-angiogenic factors	
	N3	N3	*CASS4*	*CXCL3*	–	
	N4	N4	*CTSC, HEXB, PTMA*	*PALD1, SIGLECF*	Found in healthy lung tissues, function unknown	
	N5	N5	*CCL3*	*CCL3, SIGLECF*	Found in healthy lung tissues, expressed cytokines CCL3 and CSF1, CSTB, CTSB, and IRAK2	
	–	N6	*-*	*CTSC, SIGLECF*	–	
**Mast cells**	Mast 1		*DAGLB, LTC4S, ALPI, KIT and MS4A2*		Involved in pro-tumoral and antitumoral functions, release cytokines: IL-1, IL-4, IL-8, IL-6, MCP-3, MCP-4, TNF-α, IFN-γ, LTB4, TGF-β, IL-8, VEGF, FGF-2, NGF, heparin, tryptase	(79)
	Mast 2		*CMA1, CTSG, ADCYAP1, MRO, HPGD, FUZ, MAPK6*			
**Lymphoid lineage**						
**T cell**	Helper T cells		*CD4+*		Anti-tumor response	(18, 19, 44, 89)
	Cytotoxic T cells		*CD3+, CD8+, PDCD1*		Antitumor cells, express IFNγ, Prf1(perforin), granzyme B	
	Regulatory T cells		*CD4+, FOXP3, IL2RA, CD25, CTLA-4, ENTPD1 (CD39)*		Immunosuppressive cells	
	Effector Memory T cells		*CD4+, CD8+, CD197*−*, CD45RA*−		Shows signs of dysfunction at later cancer stages,	
	Exhaustive T cells		*PD-1+, CD8+, TIM-3, CTLA-4, HLA-DR, CD38*		Characteristic of advanced-stage tumors, anti- PD1 therapy response	
	γδT cells		*TRDC+ and TRGC2+*		Cytotoxic lymphocyte, express IL17	
**B cell**	Follicular B cells		*CD20 (MS4A1), CXCR4, HLA-DR*,		Gives rise to naïve/memory B cells	(44, 114)
	Naïve B cells		*CD27*−*, IGHD+(IgD)/IGHM+(IgM)*,		Gives rise to other B cell types	
	Memory/Effector B cells		*CD27+, Granzyme B, TRAIL, MHC I, MHC II,IFNγ, IL-12*		Direct tumor cell killing, antigen presentation, macrophage polarization to M1, CD4+ T cell conversion	
	Plasma B cells		*MZB1, SDC1/CD138, PRDM1, IgG1*		Antibody secreting cells, opsonization, complement system activation, ADCC, antibody-mediated phagocytosis, antigen presentation by dendritic cells, drive cytotoxic T cell responses	
	Mucosa-associated IgA+ B cells		*IgA+, CD20−/CD19+*		Immunosuppressive, convert CD4+ T cells to Treg, promote angiogenesis, macrophage polarization, MDSC development	
	Germinal center B cells		*CCR7, GPR183, IGHM*		Undergoes class switch recombination to form other immunoglobin subtypes	
	Regulatory B cells		*PD-1, CD80, CD86*		Immunosuppressive, express inhibitory ligands and cytokines, inhibit T cell and NK cell response	
**NK cell**	CD56−		*CD16, KIR, CD57, CD69, CCR7, perforin++*		Mediates natural and antibody-dependent cellular cytotoxicity, exhibiting high levels of perforin and enhanced killing	(89, 127)
	CD56+		high*ILT2*, low*NKp46, NKG2D, NKp30, DNAM1, CD16*		Low levels of perforin, and are primarily specialized for cytokine production	
	CD56+, perforin^low^		*CD56, NKG2A, CD27, KIR*			
	CD56+, CD16^low^		*CD9, CXCR3*			
	NK-T cells		*KLRD1, FGFBP2, CX3CR1, CD8B+CD3D+*		Anti-tumorigenic in early tumor stages, show immunosuppressive activities in late-stage tumors	

### Non-Coding RNAs Regulating NK and Dendritic Cell Functions

Non-coding RNAs play significant roles in the development, maturation, and effector functions of NK cells. They directly or indirectly control the cytotoxic ability and surface expression of immune checkpoints on NK cells, thus indicating their use in antitumor therapies (128). Zhu and co-workers concluded the regulatory effect of miR-20a, which is over-expressed in various cancers. miR-20a reduces the killing-effect of NK cells to cervical cancer cells by directly targeting RUNX1 (129). Similarly, overexpression of multiple miRNAs can inhibit the cytotoxic effect of NK cells: miR-24 suppresses IFN-γ and TNF-α and thus, affecting the killing-effect of NK cells (130). miR-218-5p reduces the NK cell activity by directly targeting IL-2 secretion in lung adenocarcinoma (131). Moreover, NK cells release exosomal RNA miR-3607-3p, acting as drug targets on pancreatic cancer cells. miR-3607-3p enriched in extracellular vesicles (EVs) derived from NK cells inhibits the malignant transformation of pancreatic cancer by targeting IL-26 (132). miR-150 and miR-203 increase tumor suppression; miR-155, miR-26a/b, miR-101, miR-363, etc. lead to decreased cell survival, cell cycle progression and miR-183 and miR-1245 are known to hamper NK killing activity in TME (128). Ou and colleagues elucidated the role of miR-153 and circ_0000977 in hypoxic TME. circ_0000977 is overexpressed in pancreatic cancer and acts as a miR-153 sponge. It encourages hypoxia-mediated immune suppression by hampering NK mediated cell lysis (133). lncRNA GAS5 regulates NK cells cytotoxicity, tumor cell apoptosis, and tumor aggressiveness *via* miR-544/RUNX in liver cancer (134).

## Cells Shaping Immunotherapies

Therapeutic strategies against different cancers are advancing in drug targets and carrier agents, keeping in mind the toxicity levels and patient response. In addition to conventional therapies such as radiotherapy and chemotherapy, immunotherapy is progressing to find new immune activation and protection methods. Different forms of immunotherapy are known: cancer vaccines, cytokine therapies, adoptive cell transfer, immune checkpoint inhibitors, T/NK-cell engineering, chimeric antigen receptor (CAR) T-cell therapy, an oncolytic virus therapies (135). Advancements in single-cell heterogeneity and identification of stromal/immune cell subtypes would provide new hopes in immunotherapy as it will identify new cell receptors to targets.

### Fibroblasts as Therapeutic Agents

Diversity in CAF subtypes enlightens their immune-supportive and immune-suppressive functions *via* extensive studies of gene expression and metabolomic analysis. Targeting only the αSMA+ tumor population would not be helpful as it may result in the loss of immuno-supportive residents and may hamper significant homeostatic functions (136). But the presence of both types of the population indicates reprogramming between these two states. There is a particular need for targets that may reduce the reprogramming of fibroblasts to immune-suppressive subtypes. Targeting the immunosuppressive CAF population that promotes T cell differentiation to CD25+FOXP3+ cells and enhancing Tregs’ capacity to inhibit the proliferation of effector T cells would be an effective therapy. Remodeling these CAF subsets (CD29+ FAP++ FSP1+ αSMA++ PDGFRβ++ CAV1− DPP4+) found in aggressive cancers would directly affect T cell immunity and leads to increased CD4+ CD25+ T lymphocyte survival (33). Ford and co-workers targeted a ROS-producing enzyme NOX4 to inhibit fibroblast differentiation to CAF and found high infiltration of NK cells and CD8+ T cell to tumors, and subsequent killing of cancer cells (137). Chen and colleagues explored the relation of fibrotic TME with T cell immunotherapy. Fibrotic TME indicates poor survival and suppresses the immune response to cancer (138). Anti-FAP antibodies (sibrotuzumab), TGF-β1 (highly expressed in hypoxic tumors), cytokine therapies (IL2), CXCL12/CXCR4 signaling, angiotensin receptor blockers (ARBs), immune checkpoint blockade (ICB), T lymphocyte checkpoint inhibition, CTLA-4 directed therapy, CAR-T cells against FAP+ cells are some of the immunotherapies currently in research to target non-responsive metastatic tumors (139, 140).

### Therapeutic Aspects of Tumor Angiogenesis

Tumor cells communicate with nearby endothelial cells that line-up in angiogenic vessels and safeguard the growing tumors. Approaches to block vascular supply are long under investigation and have reached the bedside but with limited efficacy, low response rate, high resistance, and enhanced immune surveillance (141). Ongoing anti-angiogenic treatments include anti-VEGF therapies (bevacizumab), tyrosine kinase inhibitors (Sunitinib), targeting signals between cells (Revlimid), and chemotherapy combination with numerous side-effects. Decreasing tumor’s blood supply also leads to hypoxia, fuelling tumor progression, and metastasis. As an alternative therapy, vascular normalization encourages improved blood flow and reduced tumor hypoxia, understanding vascular normalization, and implementation are still in research (142). Vascular targeting would directly or indirectly enhance tumor immunity. Tumor vasculature functionality depends on the interaction with M1/M2 like TAMs, CD4+/CD8+ T cells, and other stromal/immune cells. Mpekris and co-workers highlighted the combined use of immune checkpoint and anti-angiogenetic antibodies for more favorable outcomes (143). Targeting endothelial cells (EC) subtypes is comparatively a new field and is yet to explore to find out novel anti-angiogenic candidates. Currently, combination drugs are in clinical trials that target both immune checkpoint blockades (ICBs) and angiogenesis such as Atezolizumab + bevacizumab + entinostat/Avelumab + axitinib/Atezolizumab + bevacizumab + carboplatin + pemetrexed (144).

### TAMs Targeted Therapies

TAMs are the most targeted population for immunotherapy as they constitute a major cellular component of TME. With the expansion in macrophage research, the complex phenotypic and functional properties of macrophages have unveiled their recruitment processes, polarization signals, differentiation factors, etc. Cassetta and co-workers identified 37-gene TAM signature highlighting CSF-1 and SIGLEC1 (a sialic binding receptor) mainly expressed by macrophages in aggressive human breast cancer subtypes (87). High CSF-1 and SIGLEC1 receptors in macrophages indicate tumor progression, poor clinical prognosis, and can be a targeted therapeutic option (145). Currently, TAM targeted therapies involve macrophage depletion, receptor targeting (Tyro3, Axl, and MerTK), effector functions inhibition, reprogramming towards antitumor phenotype, and inhibiting recruitment within TME. Still, all of these therapies are limited to identifying TAM-specific markers (10). Many macrophage targeted drugs are under clinical investigations: monoclonal antibodies targeting CSF-1R (IMC-CS4, R05509554, RG7155, FPA008, PLX3397) and CCL2 (Carlumab), small molecule inhibitors, antibodies targeting reprogramming strategies (anti-CD47, anti-CD40), toll-like receptor agonists, PI3Kγ inhibitors, inhibition of microRNA activity, histone deacetylase inhibitors (TMP195), anti-PD-1 and CTLA4 therapies to name a few. Each of these strategies needs careful investigations as they have certain limitations and require practicing new approaches as underpinned by current knowledge of macrophage biology.

### T Cell and B Cell Immunotherapy

T cell therapeutics is advancing since the discovery of a breakthrough of chimeric antigen receptor (CAR) -modified T cells and immune modulation using antibodies that block immune regulatory checkpoints in 2013. Both CD8+ and CD4+ cells have multifaceted roles in antitumor immunity. CD4+ cells secrete interleukin (IL)-2 activating CD8+ T cells, cytokine secretion (IFN-γ, TNF-α), activation of CD40 ligands that activates dendritic cells and leads to differentiation and maturation of B cells (146). Immune checkpoint blockade (ICB), adoptive cell transfer (ACT), and engineering TCR therapy are some primary T cell-based therapies currently in clinical trials. ICB involves the use of antibodies to neutralize inhibitory receptors CTLA-1, PD-L1/B7-H1/CD274, lymphocyte-activation protein 3 (LAG-3), T-cell immunoglobulin, mucin-domain containing-3 (TIM-3), T-cell immunoreceptor with Ig and ITIM domains (TIGIT) (147). The role of B cells in immune protection is currently in research. Besides producing antibodies, B cells have crucial roles in immune regulation or functions as an antigen-presenting cell, but involvement in cellular therapy is unclear, despite having numerous effector functions. Recently Helmink and co-workers identified differentially expressed B cell markers (*MZB1, JCHAIN, and IGLL5*) in ICBs responders relative to non-responders indicating the driving force of B cells in immunotherapy (148).

### Therapies Involving NK Cells

NK cells can induce direct killing of tumor cells, and recruit or activate of T cells and DCs. Three forms of NK therapy have emerged: Bi-specific antibodies, induction of antibody-dependent cell-mediated cytotoxicity (ADCC), and gene therapy with NK cells. Current possibilities to use NK cells are (a) targeting inhibitory NK cell surface receptors or (b) targeting activating NK cell receptors (149). NKG2A is a novel heterodimeric (expressed along with CD94) intracytoplasmic tyrosine-based inhibitory motif (ITIM) expressed on both T and NK cells and recognizes human leukocyte antigen (HLA)-E present on tumor cells (150). Binding of NKG2A/CD94 to its cognate ligand inhibits T and NK cell effector functions and thus are immunosuppressive. Andrea and co-workers developed a humanized anti-NKG2A immunoglobulin G (IgG) 4-blocking mAb (monalizumab) and showed that NKG2A blockade activates NK and CD8+ T cells (150). Another possibility is targeting activating NK cell receptors such as natural killer group 2D (NKG2D), CD16, signaling lymphocyte activation molecule (SLAM)-family members, and the natural cytotoxicity receptors (NCRs) NKp30, NKp44, and NKp46 (151). CD16 in tumor conditions binds to EGFR and leads to tumor cell proliferation. Antibody such as cetuximab and trastuzumab inhibits EGFR signaling and binds to CD16 to promote ADCC. Moreover, many tumors are consistent with a high frequency of NKp46 receptors. Targeting this receptor would help in the activation of other NK cell receptors that lead to cytokine release and the killing of tumor cells. Gauthier and co-workers developed a multifunctional antibody (NKp46 specific) targeting CD19, CD20, or EGFR as tumor antigens and triggering tumor killing by NK cells (151, 152). CD73 is another immune checkpoint that defines regulatory NK cells within TME. CD73 is associated with immune suppression and tumor progression involving CD4+ T cell suppression (153). Developing future therapies CD73 would be beneficial for T cell functioning and tumor regression.

## Conclusions

Research in the field of the tumor microenvironment is an impressive and significant stage. Despite progress in cancer immunotherapies, the incidence of tumor relapse and recurrence had necessitated a profound study of tumor heterogeneity. Clinically we are struggling with therapies that would target a wide range of cells, being unaware of subtypes that escape and show resistance. Many drugs target tumor biome by using monoclonal antibodies, angiogenic, and protein kinase inhibitors, but the precise targets are rare. With advances in single-cell technology, intra-tumoral heterogeneity is not much explored. Characterization of stromal and immune cell subtypes inhabiting at different tumor stages, early or late-stage, and the corresponding normal tissues revealed a broad range of cell subsets, differentiation patterns, and immunological regulations within TME. Targeting these well-explored immuno-modulatory cells directs the development of next-generation therapeutics. These next-generation therapies are at an early stage of development and would involve trials on small molecule inhibitors, non-coding RNAs, and cell-specific receptors that may lead to better cancer cure.

## Future Perspectives

There are still several questions in the TME that needs careful investigation. One aspect is the lineage tracing. A challenge in tumor biology is associating molecular differences among progenitor cells and their capacity to generate differentiated cell types. Studies are needed to associate changes in progenitor and mature cell types and answer questions such as a) Does a differentiated cell have the capability to transdifferentiate in other differentiated cell types in TME? If yes, what are these cells in the tumor microenvironment that shows cellular plasticity? b), and how the cellular plasticity is regulated? To answer these questions, we need to understand the origin of stromal or immune cell types in TME.

The second aspect is understanding the heterogeneity of stromal and immune cells that make up most of the TME. Fibroblasts and macrophages are among the most diversified populations whose characteristics change with their location in tumors- perivascular, hypoxic, necrotic areas, and regions of interaction with blood vessels and lymph nodes. Rare information is available on the interaction of these cells with other cell types and how different tumor zones influence the development of subtypes. Technologies such as spatial transcriptomics (seqFISH, MERFISH, STARmap) and multiplex immunofluorescence would be pivotal to identify prevailing subtypes.

The third aspect requiring attention is tumor secretome. Investigations on exosomal secretions, non-coding RNA transcriptomics, and epigenetic remodeling would boost our understanding of tumor biome. Single-cell genomic technologies are an efficient approach to understand every subtype residing within TME. It can also enhance our ability to discover novel target molecules, specific pathways targeted by these drugs, that would facilitate effective strategies. Identification of precise sub-typical population by scRNAseq analysis can serve as biomarkers to develop accessory treatments. Assessment at a single cell level can also provide us cell variations before and after immunotherapies with which medications can be optimized. In summary, we anticipate that single-cell analysis would be a great approach to understand tumor biology and designing therapies that would revolutionize the tumor treatments as it may reduce the gap between responders and non-responders.

## Author Contributions

LA and DP contributed to the study conception and design. LA collected the relevant paper and drafted the manuscript. DP revised the manuscript and supervised the study. LA and DP prepared the review figures and tables of the manuscript. All authors contributed to the article and approved the submitted version.

## Funding

This work was supported by SERB women excellence award (SB/WEA-02/2017) to DP Also, DP gratefully acknowledges financial support of IIT Ropar to the TERM laboratory.

## Conflict of Interest

The authors declare that the research was conducted in the absence of any commercial or financial relationships that could be construed as a potential conflict of interest.

## Glossary

**Table:**

ACT	adoptive cell transfer
ADCs	adult stem cells
ADCC	antibody-dependent cellular cytotoxicity
AKT	activate protein kinase B
APCs	antigen-presenting cells
apCAFs	antigen-presenting CAFs
&alpha;SMA	alpha smooth muscle actin
CAFs	cancer-associated fibroblasts
CAR-T cells	chimeric antigen receptor T cells
CAV1	Caveolin 1
cCAFs	cycling CAFs
CD	cluster of differentiation
cDC	conventional dendritic cells
circRNAs	circular RNAs
COL1A1	collagen
type I	alpha 1
COL13A1	collagen type III alpha 1 chain
COL14A1	collagen type XIV alpha 1 chain
CCL5	chemokine (C-C motif) ligand 5
CSF	colony-stimulating factor
CTLA-4	cytotoxic T-lymphocyte-associated protein 4
CXCL12	C-X-C motif chemokine 12
cyTOF	time-of-flight mass spectrometry
DC	dendritic cell
dCAFs	developmental CAFs
DPP-4	dipeptidyl peptidase-4
EC	endothelial cell
ECM	extracellular matrix
EGF	epidermal growth factor
eNOS	endothelial nitric oxide synthase 3
EMTs	epithelial-mesenchymal transitions
ERK	extracellular-signal-regulated kinase
eRNAs	enhancer RNAs
ESCs	embryonic stem cells
ESCC	esophageal squamous cell carcinoma
FAP	fibroblast activated protein
FCGR3A	low affinity immunoglobulin gamma Fc region receptor III-A
FGF	fibroblast growth factors
FGFBP-2	fibroblast growth factor binding protein 2
FOXP3	forkhead box P3
GC	germinal center
HIF-1&alpha	hypoxia-inducible factor 1-alpha
HITT	
HIF-1&alpha	inhibitor at translation level
HLA	human leukocyte antigen
iCAFs	inflammatory CAFs
Ig	immunoglobulin
IGHG1	immunoglobulin heavy constant gamma 1
JNK	c-Jun N-terminal kinase
KLF2	Kruppel-like factor 2
KLRD1	Killer cell lectin-like receptor subfamily D, member 1
LRRC15	leucine-rich repeat containing 15
lincRNAs	large intergenic non-coding RNAs
ICB	immune checkpoint blockade
IFN	interferon
IL-6	interleukin6
IL-11	interleukin 11
IL-1&beta	interleukin 1 beta
LAG3	lymphocyte-activation gene 3
LN	lymph nodes
lncRNAs	long non-coding RNAs
mAb	monoclonal antibody
MALAT1	metastasis-associated lung adenocarcinoma transcript 1
MAPK	mitogen-activated protein kinase
mCAFs	matrix CAFs
MHCII	major histocompatibility complex class II
miRNAs	MicroRNAs
MMP9	matrix metallopeptidase 9
MMTV-PyMT	mouse mammary tumor virus-polyoma middle tumor-antigen
Morrbid	myeloid RNA repressor of BCL2L11 induced death
MSCs	mesenchymal stem cells
mTOR	mammalian target of rapamycin
NCRs	natural cytotoxicity receptors
NEAT1	noncoding nuclear-enriched abundant transcript 1
NF-&kappa;B	nuclear factor kappa-light-chain-enhancer of activated B cells
NK	natural killer
NKG2D	natural killer group 2D
NOX4	NADPH oxidase 4
PDAC	pancreatic ductal adenocarcinoma
pDC	plasmacytoid DCs
PDGF	platelet-derived growth factor
PD-L1	programmed death-ligand 1
piRNAs	Piwi-interacting RNAs
PRC2	Polycomb repressor complex 2
ROS	reactive oxygen species
S100A4	S100 Calcium Binding Protein A4
scRNAseq	Single-cell RNA sequencing
SIGLEC1	sialic acid binding Ig like lectin 1
siRNAs	small interfering RNAs
SLAM	signaling lymphocyte activation molecule
sno-lincRNAs	Intron-derived small nucleolar lincRNAs
SOX-4	SRY (sex determining region Y)-box 4
STAT3	Signal transducer and activator of transcription 3
STEEL	spliced-transcript endothelial-enriched lncRNA
TAMs	tumor-associated macrophages
TCR	T cell receptor
TECs	tumor endothelial cells
TGF-&beta1	transforming growth factor beta 1
TIGIT	T cell immunoreceptor with Ig and ITIM domains
TIM-3	T-cell immunoglobulin and mucin-domain containing-3
TME	tumor microenvironment
TNF-&alpha	tumor necrosis factor alpha
TP53	Tumor protein 53
TRAIL	TNF-related apoptosis-inducing ligand
Treg	regulatory T cells
UTR	untranslated region
VEGF	vascular endothelial growth factor
vCAFs	vascular CAFs
vWF	von Willebrand factor
Wnt	Wingless-related integration site
YB-1	Y-box binding protein
ZEB1	Zinc Finger E-Box Binding Homeobox 1

## References

[1] Paget S. The distribution of secondary growths in cancer of the breast. Lancet (1889) 1:571–3. 10.1016/S0140-6736(00)49915-0 2673568

[2] Sadikovic B Al-Romaih K Squire JA Zielenska M. Cause and consequences of genetic and epigenetic alterations in human cancer. Curr Genomics (2008) 9(6):394–408. 10.2174/138920208785699580 19506729 PMC2691666

[3] Kreso A Dick JE. Evolution of the cancer stem cell model. Cell Stem Cell (2014) 14:275–91. 10.1016/j.stem.2014.02.006 24607403

[4] Srivastava D DeWitt N. In vivo cellular reprogramming: the next generation. Cell (2016) 166:1386–96. 10.1016/j.cell.2016.08.055 PMC6234007 27610565

[5] Murry CE Keller G. Differentiation of embryonic stem cells to clinically relevant populations: lessons from embryonic development. Cell (2008) 132:661–80. 10.1016/j.cell.2008.02.008 18295582

[6] Tata PR Chow RD Saladi SV Tata A Konkimalla A Bara A. Developmental history provides a roadmap for the emergence of tumor plasticity. Dev Cell (2018) 44:679–93.e5. 10.1016/j.devcel.2018.02.024 29587142 PMC5875457

[7] Waddington CH. The Strategy of Genes: A Discussion of Some Aspects of Theoretical Biology. London: Allen & Unwin (1957). 10.5694/j.1326-5377.1958.tb86500.x

[8] Jopling C Boue S Izpisua Belmonte JC. Dedifferentiation, transdifferentiation and reprogramming: three routes to regeneration. Nat Rev Mol Cell Biol (2011) 12:79–89. 10.1038/nrm3043 21252997

[9] Magnen CL Shen MM Abate-Shen C. Lineage plasticity in cancer progression and treatment. Annu Rev Cancer Biol (2018) 2:271–89. 10.1146/annurev-cancerbio-030617-050224 PMC5942183 29756093

[10] Quail DF Joyce JA. Microenvironmental regulation of tumor progression and metastasis. Nat Med (2013) 19:1423–37. 10.1038/nm.3394 PMC3954707 24202395

[11] Ren X Kang B Zhang Z. Understanding tumor ecosystems by single-cell sequencing: promises and limitations. Genome Biol (2018) 19(1):211. 10.1186/s13059-018-1593-z 30509292 PMC6276232

[12] Slack FJ Chinnaiyan AM. The role of non-coding RNAs in oncology. Cell (2019) 179:1033–55. 10.1016/j.cell.2019.10.017 PMC7347159 31730848

[13] Chen LL Yang L. Regulation of circRNA biogenesis. RNA Biol (2015) 12:381–8. 10.1080/15476286.2015.1020271 PMC4615371 25746834

[14] Bolisetty MT Graveley BR. Circuitous route to transcription regulation. Mol Cell (2013) 51:705–6. 10.1016/j.molcel.2013.09.012 PMC3839245 24074951

[15] Drápela S Bouchal J Jolly MK Culig Z Souček K. ZEB1: A Critical Regulator of Cell Plasticity, DNA Damage Response, and Therapy Resistance. Front Mol Biosci (2020) 7:36. 10.3389/fmolb.2020.00036 32266287 PMC7096573

[16] Whiteside TL. The tumor microenvironment and its role in promoting tumor growth. Oncogene (2008) 27:5904–12. 10.1038/onc.2008.271 PMC3689267 18836471

[17] Hanahan D Weinberg RA. The hallmarks of cancer. Cell (2000) 100:57–70. 10.1016/s0092-8674(00)81683-9 10647931

[18] Davidson S Efremova M Riedel A Mahata B Pramanik J Huuhtanen J. Single-Cell RNA Sequencing Reveals a Dynamic Stromal Niche That Supports Tumor Growth. Cell Rep (2020) 31(7):107628. 10.1016/j.celrep.2020.107628 32433953 PMC7242909

[19] Wagner J Rapsomaniki MA Chevrier S Anzeneder T Langwieder C Dykgers A. A Single-Cell Atlas of the Tumor and Immune Ecosystem of Human Breast Cancer. Cell (2019) 177(5):1330–45.e18. 10.1016/j.cell.2019.03.005 30982598 PMC6526772

[20] Suvà ML Tirosh I. Single-Cell RNA Sequencing in Cancer: Lessons Learned and Emerging Challenges. Mol Cell (2019) 75(1):7–12. 10.1016/j.molcel.2019.05.003 31299208

[21] Yofe I Dahan R Amit I. Single-cell genomic approaches for developing the next generation of immunotherapies. Nat Med (2020) 26(2):171–7. 10.1038/s41591-019-0736-4 32015555

[22] Kalluri R. The biology and function of fibroblasts in cancer. Nat Rev Cancer (2016) 16:582–98. 10.1038/nrc.2016.73 27550820

[23] Lambrechts D Wauters E Boeckx B Aibar S Nittner D Burton O. Phenotype molding of stromal cells in the lung tumor microenvironment. Nat Med (2018) 24(8):1277–89. 10.1038/s41591-018-0096-5 29988129

[24] Chung W Eum HH Lee HO Lee KM Lee HB Kim KT. Single-cell RNA-seq enables comprehensive tumour and immune cell profiling in primary breast cancer. Nat Commun (2017) 8:15081. 10.1038/ncomms15081 28474673 PMC5424158

[25] Hanley CJ Waise S Parker R MA L Taylor J Kimbley LM. Spatially discrete signalling niches regulate fibroblast heterogeneity in human lung cancer. bioRxiv (2020), 134270. 10.1101/2020.06.08.134270

[26] Brown FD Turley SJ. Fibroblastic reticular cells: organization and regulation of the T lymphocyte life cycle. J Immunol (2015) 194(4):1389–94. 10.4049/jimmunol.1402520 PMC4324549 25663676

[27] Bartoschek M Oskolkov N Bocci M Lovrot J Larsson C Sommarin M. Spatially and functionally distinct subclasses of breast cancer-associated fibroblasts revealed by single cell RNA sequencing. Nat Commun (2018) 9(1):5150. 10.1038/s41467-018-07582-3 30514914 PMC6279758

[28] Dominguez CX Müller S Keerthivasan S Koeppen H Hung J Gierke S. Single-Cell RNA Sequencing Reveals Stromal Evolution into LRRC15+ Myofibroblasts as a Determinant of Patient Response to Cancer Immunotherapy. Cancer Discov (2020) 10(2):232–53. 10.1158/2159-8290.CD-19-0644 31699795

[29] Elyada E Bolisetty M Laise P Flynn WF Courtois ET Burkhart RA. Cross-Species Single-Cell Analysis of Pancreatic Ductal Adenocarcinoma Reveals Antigen-Presenting Cancer-Associated Fibroblasts. Cancer Discov (2019) 9(8):1102–23. 10.1158/2159-8290.CD-19-0094 PMC6727976 31197017

[30] Kim N Kim HK Lee K Hong Y Cho JH Choi JW. Single-cell RNA sequencing demonstrates the molecular and cellular reprogramming of metastatic lung adenocarcinoma. Nat Commun (2020) 11:2285. 10.1038/s41467-020-16164-1 32385277 PMC7210975

[31] Fletcher AL Acton SE Knoblich K. Lymph node fibroblastic reticular cells in health and disease. Nat Rev Immunol (2015) 15(6):350–61. 10.1038/nri3846 PMC5152733 25998961

[32] Ohlund D Elyada E Tuveson D. Fibroblast heterogeneity in the cancer wound. J Exp Med (2014) 211:1503–23. 10.1084/jem.20140692 PMC4113948 25071162

[33] Costa A Kieffer Y Scholer-Dahirel A Pelon F Bourachot B Cardon M. Fibroblast Heterogeneity and Immunosuppressive Environment in Human Breast Cancer. Cancer Cell (2018) 33(3):463–79.e10. 10.1016/j.ccell.2018.01.011 29455927

[34] Su S Chen J Yao H Liu J Yu S Lao L. CD10+GPR77+ Cancer-Associated Fibroblasts Promote Cancer Formation and Chemoresistance by Sustaining Cancer Stemness. Cell (2018) 172(4):841–56.e16. 10.1016/j.cell.2018.01.009 29395328

[35] Mitra AK Zillhardt M Hua Y Tiwari P Murmann AE Peter ME. MicroRNAs reprogram normal fibroblasts into cancer-associated fibroblasts in ovarian cancer. Cancer Discov (2012) 2(12):1100–8. 10.1158/2159-8290.CD-12-0206 PMC3685866 23171795

[36] Wang C Gu S Cao H Li Z Xiang Z Hu K. miR-877-3p targets Smad7 and is associated with myofibroblast differentiation and bleomycin-induced lung fibrosis. Sci Rep (2016) 6:30122. 10.1038/srep30122 27444321 PMC4957095

[37] Wei P Xie Y Abel PW Huang Y Ma Q Li L. Transforming growth factor (TGF)-β1-induced miR-133a inhibits myofibroblast differentiation and pulmonary fibrosis. Cell Death Dis (2019) 10(9):670. 10.1038/s41419-019-1873-x 31511493 PMC6739313

[38] Yang X Ma L Wei R Ye T Zhou J Wen M. Twist1-induced miR-199a-3p promotes liver fibrosis by suppressing caveolin-2 and activating TGF-β pathway. Signal Transduct Target Ther (2020) 5(1):75. 10.1038/s41392-020-0169-z 32499481 PMC7272438

[39] Zhao L Ji G Le X Wang C Xu L Feng M. Long noncoding RNA LINC00092 acts in cancer-associated fibroblasts to drive glycolysis and progression of ovarian cancer. Cancer Res (2017) 77:1369–82. 10.1158/0008-5472.CAN-16-1615 28087599

[40] Tong Y Yang L Yu C Zhu W Zhou X Xiong Y. Tumor-Secreted Exosomal lncRNA POU3F3 Promotes Cisplatin Resistance in ESCC by Inducing Fibroblast Differentiation into CAFs. Mol Ther Oncolytics (2020) 18:1–13. 10.1016/j.omto.2020.05.014 32637576 PMC7321817

[41] Zhang JX Lu J Xie H Wang DP Ni HE Zhu Y. circHIPK3 regulates lung fibroblast-to-myofibroblast transition by functioning as a competing endogenous RNA. Cell Death Dis (2019) 10(3):182. 10.1038/s41419-019-1430-7 30796204 PMC6385182

[42] Blanco R Gerhardt H. VEGF and Notch in tip and stalk cell selection. Cold Spring Harb Perspect Med (2013) 3(1):a006569. 10.1101/cshperspect.a006569 23085847 PMC3530037

[43] Marcu R Choi YJ Xue J Fortin CL Wang Y Nagao RJ. Human organ-specific endothelial cell heterogeneity. iScience (2018) 4:20–35. 10.1016/j.isci.2018.05.003 30240741 PMC6147238

[44] Qian J Olbrecht S Boeckx B Vos H Laoui D Etlioglu E. A pan-cancer blueprint of the heterogeneous tumor microenvironment revealed by single-cell profiling. Cell Res (2020) 30:745–62. 10.1038/s41422-020-0355-0 PMC7608385 32561858

[45] Dudley AC. Tumor endothelial cells. Cold Spring Harb Perspect Med (2012) 2(3):a006536. 10.1101/cshperspect.a006536 22393533 PMC3282494

[46] Goveia J Rohlenova K Taverna F Treps L Conradi LC Pircher A. An integrated gene expression landscape profiling approach to identify lung tumor endothelial cell heterogeneity and angiogenic candidates. Cancer Cell (2020) 37:21–36.e13. 10.1016/j.ccell.2019.12.001 31935371

[47] Siemerink MJ Klaassen I Vogels IM Griffioen AW Van Noorden CJ Schlingemann RO. CD34 marks angiogenic tip cells in human vascular endothelial cell cultures. Angiogenesis (2012) 15(1):151–63. 10.1007/s10456-011-9251-z PMC3274677 22249946

[48] Zhao Q Eichten A Parveen A Adler C Huang Y Wang W. Single-Cell Transcriptome Analyses Reveal Endothelial Cell Heterogeneity in Tumors and Changes following Antiangiogenic Treatment. Cancer Res (2018) 78(9):2370–82. 10.1158/0008-5472.CAN-17-2728 29449267

[49] Takeda A Hollmén M Dermadi D Pan J Brulois KF Kaukonen R. Single-Cell Survey of Human Lymphatics Unveils Marked Endothelial Cell Heterogeneity and Mechanisms of Homing for Neutrophils. Immunity (2019) 51(3):561–72.e5. 10.1016/j.immuni.2019.06.027 31402260

[50] Marçola M Rodrigues CE. Endothelial progenitor cells in tumor angiogenesis: another brick in the wall. Stem Cells Int (2015) 2015:832649. 10.1155/2015/832649 26000021 PMC4427119

[51] Kalucka J de Rooij LPMH Goveia J Rohlenova K J Dumas S Meta E. Single-Cell Transcriptome Atlas of Murine Endothelial Cells. Cell (2020) 180(4):764–79.e20. 10.1016/j.cell.2020.01.015 32059779

[52] Thiriot A Perdomo C Cheng G Novitzky-Basso I McArdle S Kishimoto JK. Differential DARC/ACKR1 expression distinguishes venular from non-venular endothelial cells in murine tissues. BMC Biol (2017) 15(1):45. 10.1186/s12915-017-0381-7 28526034 PMC5438556

[53] Vanlandewijck M He L Mäe MA Andrae J Ando K Gaudio FD. A molecular atlas of cell types and zonation in the brain vasculature. Nature (2018) 554(7693):475–80. 10.1038/nature25739 29443965

[54] Maishi N Hida K. Tumor endothelial cells accelerate tumor metastasis. Cancer Sci (2017) 108(10):1921–6. 10.1111/cas.13336 PMC5623747 28763139

[55] Carmeliet P. VEGF as a key mediator of angiogenesis in cancer. Oncology (2005) 69 Suppl;3:4–10. 10.1159/000088478 16301830

[56] Zhuo H Zhao Y Cheng X Xu M Wang L Lin L. Tumor endothelial cell-derived cadherin-2 promotes angiogenesis and has prognostic significance for lung adenocarcinoma. Mol Cancer (2019) 18:34. 10.1186/s12943-019-0987-1 30832661 PMC6399986

[57] Orso F Quirico L Dettori D Coppo R Virga F Ferreira LC. Role of miRNAs in tumor and endothelial cell interactions during tumor progression. Semin Cancer Biol (2019) 60:214–24. 10.1016/j.semcancer.2019.07.024 31386907

[58] Wang Y Wang L Chen C Chu X. New Insights into the Regulatory Role of microRNA in Tumor Angiogenesis and Clinical Implications. Mol Cancer (2018) 17:22. 10.1186/s12943-018-0766-4 29415727 PMC5804051

[59] Würdinger T Tannous BA Saydam O Skog J Grau S Soutschek J. miR-296 regulates growth factor receptor overexpression in angiogenic endothelial cells. Cancer Cell (2008) 14(5):382–93. 10.1016/j.ccr.2008.10.005 PMC2597164 18977327

[60] Kong W He L Richards EJ Challa S Xu CX Permuth-Wey J. Upregulation of miRNA-155 promotes tumour angiogenesis by targeting VHL and is associated with poor prognosis and triple-negative breast cancer. Oncogene (2014) 33(6):679–89. 10.1038/onc.2012.636 PMC3925335 23353819

[61] Jiang X Wang J Deng X Xiong F Zhang S Gong Z. The role of microenvironment in tumor angiogenesis. J Exp Clin Cancer Res (2020) 39:204. 10.1186/s13046-020-01709-5 32993787 PMC7526376

[62] Goradel NH Mohammadi N Haghi-Aminjan H Farhood B Negahdari B Sahebkar A. Regulation of tumor angiogenesis by microRNAs: State of the art. J Cell Physiol (2019) 234(2):1099–110. 10.1002/jcp.27051 30070704

[63] Fang L Deng Z Shatseva T Yang J Peng C Du WW. MicroRNA miR-93 promotes tumor growth and angiogenesis by targeting integrin-β8. Oncogene (2011) 30:806–21. 10.1038/onc.2010.465 20956944

[64] Ye J Wu X Wu D Wu P Ni C Zhang Z. miRNA-27b targets vascular endothelial growth factor C to inhibit tumor progression and angiogenesis in colorectal cancer. PLoS One (2013) 8(4):e60687. 10.1371/journal.pone.0060687 23593282 PMC3625233

[65] Kopp F Mendell JT. Functional Classification and Experimental Dissection of Long Noncoding RNAs. Cell (2018) 172(3):393–407. 10.1016/j.cell.2018.01.011 29373828 PMC5978744

[66] Yuan SX Yang F Yang Y Tao QF Zhang J Huang G. Long noncoding RNA associated with microvascular invasion in hepatocellular carcinoma promotes angiogenesis and serves as a predictor for hepatocellular carcinoma patients’ poor recurrence-free survival after hepatectomy. Hepatology (2012) 56(6):2231–41. 10.1002/hep.25895 22706893

[67] Man HSJ Sukumar AN Lam GC Turgeon PJ Yan MS Ku KH. Angiogenic patterning by STEEL, an endothelial-enriched long noncoding RNA. Proc Natl Acad Sci U S A (2018) 115:2401–6. 10.1073/pnas.1715182115 PMC5877935 29467285

[68] Yu B Wang S. Angio-LncRs: LncRNAs that regulate angiogenesis and vascular disease. Theranostics (2018) 8(13):3654–75. 10.7150/thno.26024 PMC6037039 30026873

[69] Wang X Li L Zhao K Lin Q Li H Xue X. A Novel LncRNA HITT Forms a Regulatory Loop with HIF-1alpha to Modulate Angiogenesis and Tumor Growth. Cell Death Differ (2020) 27:1431–46. 10.1038/s41418-019-0449-8 PMC7205893 31700144

[70] Yang W Du WW Li X Yee AJ Yang BB. Foxo3 activity promoted by non-coding effects of circular RNA and Foxo3 pseudogene in the inhibition of tumor growth and angiogenesis. Oncogene (2016) 35(30):3919–31. 10.1038/onc.2015.460 26657152

[71] Li CY Ma L Yu B. Circular RNA hsa_circ_0003575 regulates oxLDL induced vascular endothelial cells proliferation and angiogenesis. BioMed Pharmacother (2017) 95:1514–9. 10.1016/j.biopha.2017.09.064 28946214

[72] Liu H Ma X Mao Z Liu H Shen M Zhu J. Circular RNA has_circ_0003204 inhibits oxLDL-induced vascular endothelial cell proliferation and angiogenesis. Cell Signal (2020) 70:109595. 10.1016/j.cellsig.2020.109595 32151659

[73] Chen C Huang Z Mo X Song Y Li X Li X. The circular RNA 001971/miR-29c-3p axis modulates colorectal cancer growth, metastasis, and angiogenesis through VEGFA. J Exp Clin Cancer Res (2020) 39(1):91. 10.1186/s13046-020-01594-y 32430042 PMC7236474

[74] Ji X Shan L Shen P He M. Circular RNA circ_001621 promotes osteosarcoma cells proliferation and migration by sponging miR-578 and regulating VEGF expression. Cell Death Dis (2020) 11(1):18. 10.1038/s41419-019-2204-y 31907361 PMC6944700

[75] Gao S Yu Y Liu L Meng J Li G. Circular RNA hsa_ circ_0007059 restrains proliferation and epithelial-mesenchymal transition in lung cancer cells via inhibiting microRNA-378. Life Sci (2019) 233:116692. 10.1016/j.lfs.2019.116692 31351967

[76] Castellano JJ Navarro A Vinolas N Marrades RM Moises J Cordeiro A. LincRNA-p21 Impacts Prognosis in Resected Non-Small Cell Lung Cancer Patients through Angiogenesis Regulation. J Thorac Oncol (2016) 11(12):2173–82. 10.1016/j.jtho.2016.07.015 27496652

[77] Shimasaki N Jain A Campana D. NK cells for cancer immunotherapy. Nat Rev Drug Discov (2020) 19(3):200–18. 10.1038/s41573-019-0052-1 31907401

[78] Teijeira Á Garasa S Gato M Alfaro C Migueliz I Cirella A. CXCR1 and CXCR2 Chemokine Receptor Agonists Produced by Tumors Induce Neutrophil Extracellular Traps that Interfere with Immune Cytotoxicity. Immunity (2020) 52(5):856–71.e8. 10.1016/j.immuni.2020.03.001 32289253

[79] Zilionis R Engblom C Pfirschke C Savova V Zemmour D Saatcioglu HD. Single-Cell Transcriptomics of Human and Mouse Lung Cancers Reveals Conserved Myeloid Populations across Individuals and Species. Immunity (2019) 50(5):1317–34.e10. 10.1016/j.immuni.2019.03.009 30979687 PMC6620049

[80] Chen H Ye F Guo G. Revolutionizing immunology with single-cell RNA sequencing. Cell Mol Immunol (2019) 16(3):242–9. 10.1038/s41423-019-0214-4 PMC6460502 30796351

[81] Gao M Ling M Tang X Wang S Xiao X Qiao Y. Comparison of high-throughput single-cell RNA sequencing data processing pipelines. Briefings Bioinf (2020) bbaa116. 10.1093/bib/bbaa116 34020539

[82] Villani AC Satija R Reynolds G Sarkizova S Shekhar K Fletcher J. Single-cell RNA-seq reveals new types of human blood dendritic cells, monocytes, and progenitors. Science (2017) 356(6335):eaah4573. 10.1126/science.aah4573 28428369 PMC5775029

[83] Kapellos TS Bonaguro L Gemünd I Reusch N Saglam A Hinkley ER. Human Monocyte Subsets and Phenotypes in Major Chronic Inflammatory Diseases. Front Immunol (2019) 10:2035. 10.3389/fimmu.2019.02035 31543877 PMC6728754

[84] De Palma M Murdoch C Venneri MA Naldini L Lewis CE. Tie2-expressing monocytes: regulation of tumor angiogenesis and therapeutic implications. Trends Immunol (2007) 28(12):519–24. 10.1016/j.it.2007.09.004 17981504

[85] Devalaraja S To TKJ Folkert IW Natesan R Alam Z Li M. Tumor-Derived Retinoic Acid Regulates Intratumoral Monocyte Differentiation to Promote Immune Suppression. Cell (2020) 180(6):1098–114.e16. 10.1016/j.cell.2020.02.042 32169218 PMC7194250

[86] Mantovani A Locati M. Tumor-associated macrophages as a paradigm of macrophage plasticity, diversity, and polarization: lessons and open questions. Arterioscler Thromb Vasc Biol (2013) 33(7):1478–83. 10.1161/ATVBAHA.113.300168 23766387

[87] Cassetta L Fragkogianni S Sims AH Swierczak A Forrester LM Zhang H. Human Tumor-Associated Macrophage and Monocyte Transcriptional Landscapes Reveal Cancer-Specific Reprogramming, Biomarkers, and Therapeutic Targets. Cancer Cell (2019) 35(4):588–602.e10. 10.1016/j.ccell.2019.02.009 30930117 PMC6472943

[88] Thorsson V Gibbs DL Brown SD Wolf D Bortone DS Ou Yang TH. The immune landscape of cancer. Immunity (2018) 48:812–30.e14. 10.1016/j.immuni.2018.03.023 29628290 PMC5982584

[89] Azizi E Carr AJ Plitas G Cornish AE Konopacki C Prabhakaran S. Single-Cell Map of Diverse Immune Phenotypes in the Breast Tumor Microenvironment. Cell (2018) 174(5):1293–308.e36. 10.1016/j.cell.2018.05.060 29961579 PMC6348010

[90] Curtale G Rubino M Locati M. MicroRNAs as Molecular Switches in Macrophage Activation. Front Immunol (2019) 10:799. 10.3389/fimmu.2019.00799 31057539 PMC6478758

[91] Squadrito ML Etzrodt M De Palma M Pittet MJ. MicroRNA-mediated control of macrophages and its implications for cancer. Trends Immunol (2013) 34:350–9. 10.1016/j.it.2013.02.003 PMC3700601 23498847

[92] Yang D Liu K Fan L Liang W Xu T Jiang W. LncRNA RP11-361F15.2 promotes osteosarcoma tumorigenesis by inhibiting M2-Like polarization of tumor-associated macrophages of CPEB4. Cancer Lett (2020) 473:33–49. 10.1016/j.canlet.2019.12.041 31904478

[93] Liang Y Song X Li Y Chen B Zhao W Wang L. LncRNA BCRT1 promotes breast cancer progression by targeting miR-1303/PTBP3 axis. Mol Cancer (2020) 19(1):85. 10.1186/s12943-020-01206-5 32384893 PMC7206728

[94] Liang Z Liu H Wang F Xiong L Zhou C Hu T. LncRNA RPPH1 promotes colorectal cancer metastasis by interacting with TUBB3 and by promoting exosomes-mediated macrophage M2 polarization. Cell Death Dis (2019) 10:829. 10.1038/s41419-019-2077-0 31685807 PMC6828701

[95] Gao Y Fang P Li WJ Zhang J Wang GP Jiang DF. LncRNA NEAT1 sponges miR-214 to regulate M2 macrophage polarization by regulation of B7-H3 in multiple myeloma. Mol Immunol (2020) 117:20–8. 10.1016/j.molimm.2019.10.026 31731055

[96] Li X Lei Y Wu M Li N. Regulation of Macrophage Activation and Polarization by HCC-Derived Exosomal lncRNA TUC339. Int J Mol Sci (2018) 19:2958. 10.3390/ijms19102958 PMC6213212 30274167

[97] Yi C Xiong JB Zhang GY Liu Y Jie ZG Li ZR. Long Noncoding RNA UCA1 Regulates PRL-3 Expression by Sponging MicroRNA-495 to Promote the Progression of Gastric Cancer. Mol Ther Nucleic Acids (2020) 196:853–64. 10.1016/j.omtn.2019.10.020 PMC6992896 31982772

[98] Golubovskaya V Wu L. Different Subsets of T Cells, Memory, Effector Functions, and CAR-T Immunotherapy. Cancers (2016) 8(3):36. 10.3390/cancers8030036 PMC4810120 26999211

[99] Kaech SM Cui W. Transcriptional control of effector and memory CD8+ T cell differentiation. Nat Rev Immunol (2012) 12(11):749–61. 10.1038/nri3307 PMC4137483 23080391

[100] Curiel TJ Coukos G Zou L Alvarez X Cheng P Mottram P. Specific recruitment of regulatory T cells in ovarian carcinoma fosters immune privilege and predicts reduced survival. Nat Med (2004) 10:942–9. 10.1038/nm1093 15322536

[101] Ji Y Fioravanti J Zhu W Wang H Wu T Hu J. miR-155 harnesses Phf19 to potentiate cancer immunotherapy through epigenetic reprogramming of CD8+ T cell fate. Nat Commun (2019) 10:2157. 10.1038/s41467-019-09882-8 31089138 PMC6517388

[102] Li Q Johnston N Zheng X Wang H Zhang X Gao D. miR-28 modulates exhaustive differentiation of T cells through silencing programmed cell death-1 and regulating cytokine secretion. Oncotarget (2016) 7:53735–50. 10.18632/oncotarget.10731 PMC5288217 27447564

[103] Wu H Neilson JR Kumar P Manocha M Shankar P Sharp PA. miRNA Profiling of Naïve, Effector and Memory CD8 T Cells. PLoS One (2007) 2(10):e1020. 10.1371/journal.pone.0001020 17925868 PMC2000354

[104] Ye Z Li G Kim C Hu B Jadhav RR Weyand CM. Regulation of miR-181a expression in T cell aging. Nat Commun (2018) 9:3060. 10.1038/s41467-018-05552-3 30076309 PMC6076328

[105] Min S Li L Zhang M Zhang Y Liang X Xie Y. TGF-β-associated miR-27a inhibits dendritic cell-mediated differentiation of Th1 and Th17 cells by TAB3, p38 MAPK, MAP2K4 and MAP2K7. Genes Immun (2012) 13:621–31. 10.1038/gene.2012.45 23034448

[106] Xu S Tao Z Hai B Liang H Shi Y Wang T. miR-424(322) reverses chemoresistance via T-cell immune response activation by blocking the PD-L1 immune checkpoint. Nat Commun (2016) 7:11406. 10.1038/ncomms11406 27147225 PMC4858750

[107] Kotzin JJ Iseka F Wright J Basavappa MG Clark ML Ali MA. The long noncoding RNA Morrbid regulates CD8 T cells in response to viral infection. Proc Natl Acad Sci U S A (2019) 116(24):11916–25. 10.1073/pnas.1819457116 PMC6575676 31138702

[108] Wang J Zhao X Wang Y Ren F Sun D Yan Y. circRNA-002178 act as a ceRNA to promote PDL1/PD1 expression in lung adenocarcinoma. Cell Death Dis (2020) 11:32. 10.1038/s41419-020-2230-9 31949130 PMC6965119

[109] Varn FS Mullins DW Arias-Pulido H Fiering S Cheng C. Adaptive immunity programmes in breast cancer. Immunology (2016) 150(1):25–34. 10.1111/imm.12664 27564847 PMC5341497

[110] Wang S Liu W Ly D Xu H Qu L Zhang L. Tumor-infiltrating B cells: their role and application in anti-tumor immunity in lung cancer. Cell Mol Immunol (2019) 16:6–18. 10.1038/s41423-018-0027-x 29628498 PMC6318290

[111] Schoorl R Riviere AB Borne AE Feltkamp-Vroom TM. Identification of T and B lymphocytes in human breast cancer with immunohistochemical techniques. Am J Pathol (1976) 84:529–44. 10.1038/s41577-019-0257-x PMC2032535 183507

[112] Nielsen JS Sahota RA Milne K Kost SE Nesslinger NJ Watson PH. CD20+ tumor-infiltrating lymphocytes have an atypical CD27- memory phenotype and together with CD8+ T cells promote favourable prognosis in ovarian cancer. Clin Cancer Res (2012) 18:3281–92. 10.1158/1078-0432.CCR-12-0234 22553348

[113] Takemori T Kaji T Takahashi Y Shimoda M Rajewsky K. Generation of memory B cells inside and outside germinal centers. Eur J Immunol (2014) 44:1258–64. 10.1002/eji.201343716 24610726

[114] Sharonov GV Serebrovskaya EO Yuzhakova DV Britanova OV Chudakov DM. B cells, plasma cells and antibody repertoires in the tumour microenvironment. Nat Rev Immunol (2020) 20(5):294–307. 10.1038/s41577-019-0257-x 31988391

[115] Zhang C Xin H Zhang W Yazaki PJ Zhang Z Le K. CD5 Binds to Interleukin-6 and Induces a Feed-Forward Loop with the Transcription Factor STAT3 in B Cells to Promote Cancer. Immunity (2016) 44(4):913–23. 10.1016/j.immuni.2016.04.003 PMC4844010 27096320

[116] Tsou P Katayama H Ostrin EJ Hanash SM. The Emerging Role of B Cells in Tumor Immunity. Cancer Res (2016) 76(19):5597–601. 10.1158/0008-5472.CAN-16-0431 27634765

[117] Li J Wan Y Ji Q Fang Y Wu Y. The role of microRNAs in B-cell development and function. Cell Mol Immunol (2013) 10:107–12. 10.1038/cmi.2012.62 PMC4003047 23314697

[118] Paladini L Fabris L Bottai G Raschioni C Calin GA Santarpia L. Targeting microRNAs as key modulators of tumor immune response. J Exp Clin Cancer Res (2016) 35:103. 10.1186/s13046-016-0375-2 27349385 PMC4924278

[119] Liu R Lu Z Gu J Liu J Huang E Liu X. MicroRNAs 15A and 16-1 Activate Signaling Pathways That Mediate Chemotaxis of Immune Regulatory B cells to Colorectal Tumors. Gastroenterology (2018) 154(3):637–51.e7. 10.1053/j.gastro.2017.09.045 29031499

[120] Agirre X Meydan C Jiang Y Garate L Doane AS Li Z. Long non-coding RNAs discriminate the stages and gene regulatory states of human humoral immune response. Nat Commun (2019) 10:821. 10.1038/s41467-019-08679-z 30778059 PMC6379396

[121] Zhou M Zhang Z Bao S Hou P Yan C Su J. Computational recognition of lncRNA signature of tumor-infiltrating B lymphocytes with potential implications in prognosis and immunotherapy of bladder cancer (published online ahead of print, 2020 May 8). Brief Bioinform (2020) bbaa047. 10.1093/bib/bbaa047 32382761

[122] Pyfrom SC Quinn CC Dorando HK Luo H Payton JE. BCALM (AC099524.1) Is a Human B Lymphocyte-Specific Long Noncoding RNA That Modulates B Cell Receptor-Mediated Calcium Signaling. J Immunol (2020) 205(3):595–607. 10.4049/jimmunol.2000088 32571842 PMC7372127

[123] Petri A Dybkær K Bøgsted M Thrue CA Hagedron PH Schmitz A. Long Noncoding RNA Expression during Human B-Cell Development. PLoS One (2015) 10(9):e0138236. 10.1371/journal.pone.0138236 26394393 PMC4578992

[124] Poli A Michel T Thérésine M Andrès E Hentges F Zimmer J. CD56bright natural killer (NK) cells: an important NK cell subset. Immunology (2009) 126(4):458–65. 10.1111/j.1365-2567.2008.03027.x PMC2673358 19278419

[125] Cremer I Fridman WH Sautes-Fridman C. Tumor microenvironment in NSCLC suppresses NK cells function. Oncoimmunology (2012) 1:244–6. 10.4161/onci.1.2.18309 PMC3377004 22720258

[126] Masucci MT Minopoli M Carriero MV. Tumor Associated Neutrophils. Their Role in Tumorigenesis, Metastasis, Prognosis and Therapy. Front Oncol (2019) 9:1146. 10.3389/fonc.2019.01146 31799175 PMC6874146

[127] Stabile H Fionda C Gismondi A Santoni A. Role of Distinct Natural Killer Cell Subsets in Anticancer Response. Front Immunol (2017) 8:293. 10.3389/fimmu.2017.00293 28360915 PMC5352654

[128] Pesce S Greppi M Ferretti E Obino V Carlomagno S Rutigliani M. miRNAs in NK Cell-Based Immune Responses and Cancer Immunotherapy. Front Cell Dev Biol (2020) 8:119. 10.3389/fcell.2020.00119 32161759 PMC7053181

[129] Zhu SY Wu QY Zhang CX Wang Q Ling J Huang XT. miR-20a inhibits the killing effect of natural killer cells to cervical cancer cells by downregulating RUNX1. Biochem Biophys Res Commun (2018) 505(1):309–16. 10.1016/j.bbrc.2018.09.102 30249397

[130] Zhang LL Zhang LF Shi YB. miR-24 inhibited the killing effect of natural killer cells to colorectal cancer cells by downregulating Paxillin. BioMed Pharmacother (2018) 101:257–63. 10.1016/j.biopha.2018.02.024 29494963

[131] Yang Q Li J Hu Y Tang X Yu L Dong L. MiR-218-5p Suppresses the Killing Effect of Natural Killer Cell to Lung Adenocarcinoma by Targeting SHMT1. Yonsei Med J (2019) 60(6):500–8. 10.3349/ymj.2019.60.6.500 PMC6536398 31124332

[132] Sun H Shi K Qi K Kong H Zhang J Dai S. Natural Killer Cell-Derived Exosomal miR-3607-3p Inhibits Pancreatic Cancer Progression by Targeting IL-26. Front Immunol (2019) 10:2819. 10.3389/fimmu.2019.02819 31921112 PMC6918866

[133] Ou ZL Luo Z Wei W Liang S Gao TL Lu YB. Hypoxia-induced shedding of MICA and HIF1A-mediated immune escape of pancreatic cancer cells from NK cells: role of circ_0000977/miR-153 axis. RNA Biol (2019) 16(11):1592–603. 10.1080/15476286.2019.1649585 PMC6779391 31402756

[134] Fang P Xiang L Chen W Li S Huang S Li J. LncRNA GAS5 enhanced the killing effect of NK cell on liver cancer through regulating miR-544/RUNX3. Innate Immun (2019) 25(2):99–109. 10.1177/1753425919827632 30774011 PMC6830859

[135] Zhang Y Zhang Z. The history and advances in cancer immunotherapy: understanding the characteristics of tumor-infiltrating immune cells and their therapeutic implications. Cell Mol Immunol (2020) 17:807–21. 10.1038/s41423-020-0488-6 PMC7395159 32612154

[136] Chauhan VP Chen IX Tong R Ng MR Martin JD Naxerova K. Reprogramming the microenvironment with tumor-selective angiotensin blockers enhances cancer immunotherapy. Proc Natl Acad Sci U S A (2019) 116(22):10674–80. 10.1073/pnas.1819889116 PMC6561160 31040208

[137] Ford K Hanley CJ Mellone M Szyndralewiez C Heitz F Wiesel P. NOX4 Inhibition Potentiates Immunotherapy by Overcoming Cancer-Associated Fibroblast-Mediated CD8 T-cell Exclusion from Tumors. Cancer Res (2020) 80(9):1846–60. 10.1158/0008-5472.CAN-19-3158 PMC7611230 32122909

[138] Chen X Song E. Turning foes to friends: targeting cancer-associated fibroblasts. Nat Rev Drug Discov (2019) 18(2):99–115. 10.1038/s41573-018-0004-1 30470818

[139] Lee J Fassnacht M Nair S Boczkowski D Gilboa E. Tumor immunotherapy targeting fibroblast activation protein, a product expressed in tumor-associated fibroblasts. Cancer Res (2005) 65(23):11156–63. 10.1158/0008-5472.CAN-05-2805 16322266

[140] Feig C Jones JO Kraman M Wells RJB Deonarine A Chan DS. Targeting CXCL12 from FAP-expressing carcinoma-associated fibroblasts synergizes with anti-PD-L1 immunotherapy in pancreatic cancer. Proc Natl Acad Sci U S A (2013) 110(50):20212–7. 10.1073/pnas.1320318110 PMC3864274 24277834

[141] Carmeliet P Jain R. Molecular mechanisms and clinical applications of angiogenesis. Nature (2011) 473:298–307. 10.1038/nature10144 21593862 PMC4049445

[142] Wong PP Bodrug N Hodivala-Dilke KM. Exploring Novel Methods for Modulating Tumor Blood Vessels in Cancer Treatment. Curr Biol (2016) 26:R1161–6. 10.1016/j.cub.2016.09.043 27825457

[143] Mpekris F Voutouri C Baish JW Duda DG Munn LL Stylianopoulos T. Combining microenvironment normalization strategies to improve cancer immunotherapy. Proc Natl Acad Sci (2020) 117:(7)3728–3737. 10.1073/pnas.1919764117 PMC7035612 32015113

[144] Yi M Jiao D Qin S Chu Q Wu K Li A. Synergistic effect of immune checkpoint blockade and anti–angiogenesis in cancer treatment. Mol Cancer (2019) 18:60. 10.1186/s12943-019-0974-6 30925919 PMC6441150

[145] Zhang Y Li JQ Jiang ZZ Li L Wu Y Zheng L. CD169 identifies an anti-tumour macrophage subpopulation in human hepatocellular carcinoma. J Pathol (2016) 239:231–41. 10.1002/path.4720 27174787

[146] Tay NQ Lee DCP Chua YL Prabhu N Gascoigne NRJ Kemeny DM. CD40L Expression Allows CD8+ T Cells to Promote Their Own Expansion and Differentiation through Dendritic Cells. Front Immunol (2017) 8:1484. 10.3389/fimmu.2017.01484 29163545 PMC5672143

[147] Akinleye A Rasool Z. Immune checkpoint inhibitors of PD-L1 as cancer therapeutics. J Hematol Oncol (2019) 12:92. 10.1186/s13045-019-0779-5 31488176 PMC6729004

[148] Helmink BA Reddy SM Gao J Zhang S Basar R Thakur R. B cells and tertiary lymphoid structures promote immunotherapy response. Nature (2020) 577:549–55. 10.1038/s41586-019-1922-8 PMC8762581 31942075

[149] Erpenbeck L Schön MP. Neutrophil extracellular traps: protagonists of cancer progression? Oncogene (2017) 36(18):2483–90. 10.1038/onc.2016.406 27941879

[150] André P Denis C Soulas C Caillet CB Lopez J Arnoux T. Anti-NKG2A mAb Is a Checkpoint Inhibitor that Promotes Anti-tumor Immunity by Unleashing Both T and NK Cells. Cell (2018) 175(7):1731–43.e13. 10.1016/j.cell.2018.10.014 30503213 PMC6292840

[151] Gauthier L Morel A Anceriz N Rossi B Alvarez AB Grondin G. Multifunctional Natural Killer Cell Engagers Targeting NKp46 Trigger Protective Tumor Immunity. Cell (2019) 177(7):1701–13.e16. 10.1016/j.cell.2019.04.041 31155232

[152] Terrén I Orrantia A Mikelez-Alonso I Vitallé J Zenarruzabeitia O Borrego F. NK Cell-Based Immunotherapy in Renal Cell Carcinoma. Cancers (Basel) (2020) 12(2):316. 10.3390/cancers12020316 PMC7072691 32013092

[153] Neo SY Yang Y Record J Ma R Chen X Chen Z. CD73 immune checkpoint defines regulatory NK cells within the tumor microenvironment. J Clin Invest (2020) 130(3):1185–98. 10.1172/JCI128895 PMC7269592 31770109

